# Molecular Recognition of Steroids and Bile Salts by Substituted Cyclodextrins: The Influence of Gonane Isomerism and Rim Functionalization on Complexation Thermodynamics

**DOI:** 10.3390/molecules31172999

**Published:** 2026-08-27

**Authors:** Andrea Usenik, Stella Katovčić, Josip Požar

**Affiliations:** Department of Chemistry, University of Zagreb Faculty of Science, Horvatovac 102A, HR-10000 Zagreb, Croatia; skatovcic.chem@pmf.hr

**Keywords:** cyclodextrin, substituted cyclodextrin, steroid, bile salt, isothermal titration calorimetry, enthalpy–entropy compensation, hydrophobic effect

## Abstract

Rim functionalization is what makes cyclodextrins pharmaceutically usable, yet how the type and degree of substitution influence steroid recognition has been established almost solely for conjugated bile salts. Using isothermal titration calorimetry and ^1^H NMR, we determined the complexation thermodynamics of neutral androsterone, its *cis*-isomer etiocholanolone, and the cholate, deoxycholate, and lithocholate anions with 2-hydroxypropyl-β- and -γ-, randomly methylated β-, and sulfobutylether-β-cyclodextrins, all compared with the parent hosts measured under identical conditions. Two findings stand out. First, unlike β-cyclodextrin, its derivatives bind the steroids exclusively as 1:1 complexes regardless of gonane isomerism. Second, in sulfobutylether hosts, the degree of substitution could be used as a selectivity filter: affinity for the most polar cholate falls steeply once degree of substitution (DS) exceeds about six, while binding of the least hydroxylated lithocholate is barely affected, opposite to the loss of selectivity usually reported upon substitution. For all hosts, functionalization lowers stability and shifts binding toward the classical hydrophobic effect. The obtained results, apart from the type of substituent, identify the host substitution degree as a potential lever for choosing cyclodextrin excipients that defy bile salt competition. Moreover, the substituted β-cyclodextrins do not form sparingly soluble complexes with *trans*-steroids, as does the parent receptor.

## 1. Introduction

Steroids are a class of biorelevant compounds present in all three biological kingdoms, where they act as structural and signaling constituents of membranes and hormones, and participate in the digestion of fats [1]. Their synthetic derivatives are widely used as anti-inflammatory [2] and antimicrobial [3] drugs, hormone substitutes and, not least, contraceptives [4]. The tetracyclic gonane core is markedly hydrophobic, to the extent that even the more polar members of the family (bile salts) exhibit at best millimolar aqueous solubility, which significantly reduces steroid bioavailability. Complex formation with cyclodextrins (CDs) provides an effective means of overcoming this limitation. Namely, these natural cyclic glucose oligomers possess hydrophobic cavities, while hydroxyl (OH) groups line the solvent-exposed surface and the receptor portals, making larger members of the family (β-cyclodextrin and γ-cyclodextrin) efficient steroid carriers in aqueous media [5,6,7,8]. Conversely, **β-CD** forms sparingly soluble 1:2 (guest:host) complexes with cholesterol [9,10], enabling its removal from dairy products and fats [11,12,13]. Of particular relevance, bile salt anions can compete with pharmaceutically active ingredients for the binding sites of β- and γ-cyclodextrins, thereby affecting drug absorption kinetics and in vivo performance [14,15,16].

Relying on relevant literature data, in our recent study, we examined in detail how gonane isomerism and receptor size govern the stoichiometry and thermodynamics of neutral steroid and bile salt anion inclusion by the parent cyclodextrins [17]. Two findings are crucial in the context of the research herein presented. First, steroid isomerism dictates the binding stoichiometry with **β-CD**: quasi-planar *trans*-steroids (androsterone (**A**) and testosterone) form both 1:1 and 1:2 (guest:host) complexes, whereas the V-shaped *cis*-isomer etiocholanolone (**E**) forms a 1:1 complex (Table 1) exclusively. Second, the inclusion thermodynamic fingerprint is defined by the cavity size: the incorporation into **β-CD** is enthalpy-driven and entropy-opposed (non-classical hydrophobic effect), while the larger **γ-CD** binds the same guests through a dominant favorable entropic term (classical hydrophobic effect). Despite the notable differences in the binding thermodynamics, the examined neutral guests form complexes of comparable stability with both cyclodextrins (4.5 < log *K°* < 5.2, Table 1). Apart from that, we established that the affinity of **β-CD** and **γ-CD** for bile salt anions decreases with hydroxylation of the gonane backbone.

Although the described results bear significance for application-oriented research, native cyclodextrins are known to be of limited pharmaceutical use. Due to extensive intra- and intermolecular hydrogen bonding, the seven-membered **β-CD** is quite sparingly soluble and can be nephrotoxic upon prolonged parenteral administration [18,19]. Compared with its smaller analogue, **γ-CD** displays an approximately tenfold higher aqueous solubility and does not induce renal damage upon administration; nevertheless, its solubility can be further improved by functionalization. The most widely used 2-hydroxypropylated (**HP**), sulfobutylether (**SB**), and randomly methylated (**RM**) derivatives of **β-CD** feature higher aqueous solubility than the parent compound by orders of magnitude [20,21], are among the most widely used parenteral solubilizers [22,23,24,25,26,27], and are approved as excipients in numerous marketed formulations [28,29,30]. Likewise, 2-hydroxypropyl-γ-cyclodextrin (**HP-γ-CD**) is often preferred as an excipient over the natural analogue and is the most commonly used **γ-CD** derivative in the pharmaceutical industry [31,32,33].

Apart from increasing aqueous solubility, the introduced substituents can influence the organization of surrounding water molecules and, to some extent, hydration of the cavity, thereby modifying the receptor properties of cyclodextrins. On the other hand, anionic and cationic functionalities can engage in electrostatic interactions with the charged guest, which can be either beneficial or detrimental to complexation. Schönbeck et al. [34,35] reported that enthalpy and entropy of bile salt anion complexation with hydroxypropylated **β-CD** exhibited a strong linear increase with the receptor substitution degree, leading to enthalpy–entropy compensation. In a subsequent study [36] involving methylated **β-CD**, a similar trend was observed up to a certain substitution degree, above which both Δ_r_*H*° and Δ_r_*S*° started to decrease, an effect linked to distortion of the cavity at high substitution [37]. Interestingly, the negatively charged sulfobutylether-**β-CD**, comprising approximately seven substituents, exhibited very similar affinity towards tauro- and glycoconjugated bile salt anions as the native cyclodextrin [38,39].

Concerning the complexation of steroids with modified γ-cyclodextrins, the binding of bile salts with methyl, sulfobutylether, and 2-hydroxypropyl derivatives and the native compound has been studied so far [40]. Overall, rim functionalization decreased complex stability, the effect being more pronounced for sulfobutylether and hydroxypropyl than for methyl substituents; however, fully methylated **γ-CD** did not bind the anions. As in the case of smaller analogues, pronounced dependence of the reaction enthalpy and entropy on the degree of substitution was observed, resulting in almost substituent-invariant Δ_r_*G*° [40,41].

**Figure 1:**
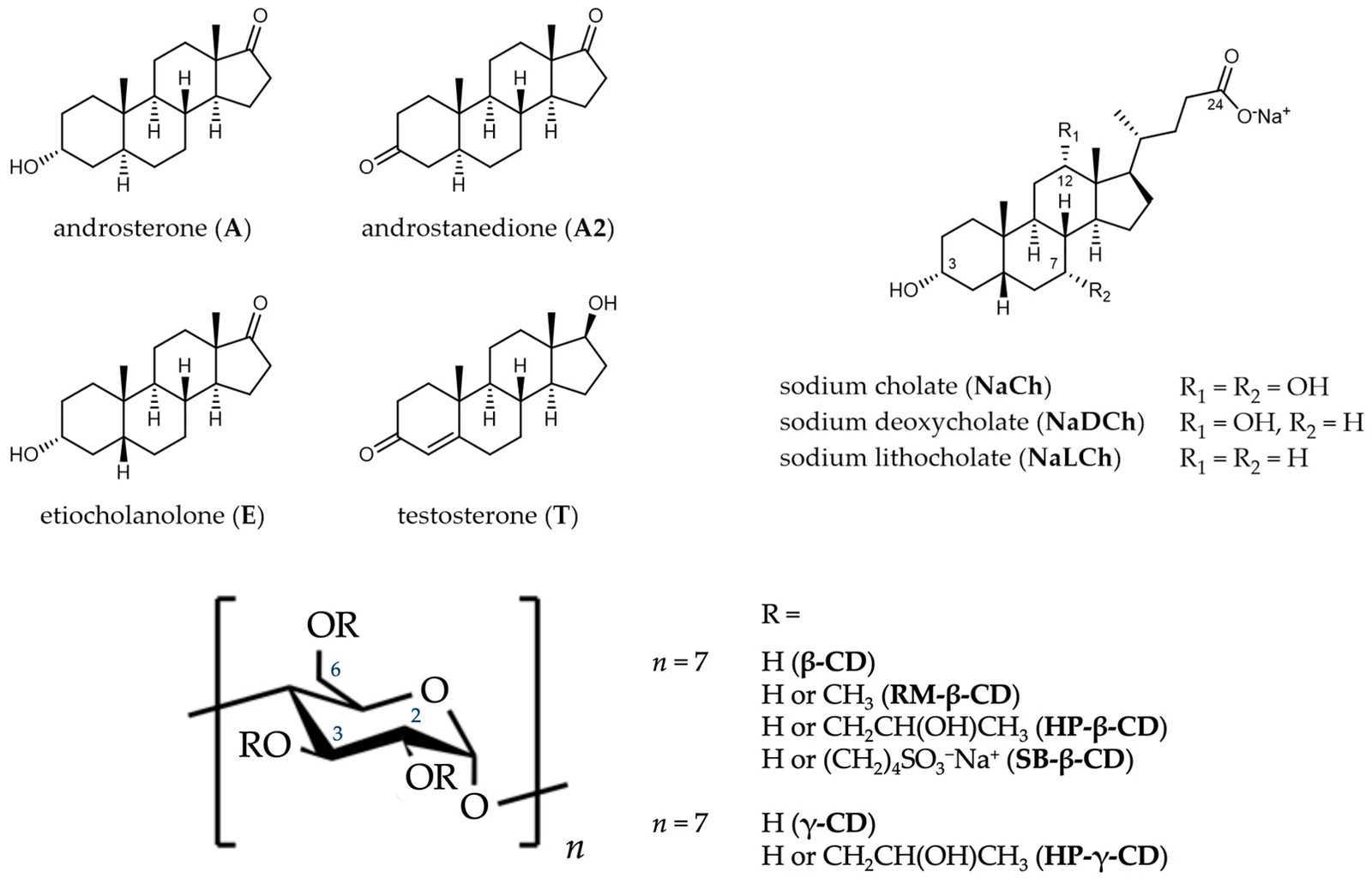
Structures of the steroids, bile salts, and cyclodextrin derivatives investigated herein and in our previous companion study (ref. [17]). The average degree of cyclodextrin substitution (DS) is given as the average number of substituents per cyclodextrin molecule.

Comparable calorimetric data for the neutral steroids are, to our knowledge, lacking and, importantly, all studied bile salt anions are *cis*-gonane isomers. Building on our companion study [17] and the mentioned literature data, we now examine the effect of rim functionalization on the inclusion of androsterone (**A**), its *cis*-isomer etiocholanolone (**E**), and three bile salt anions: cholate (**Ch^−^**), deoxycholate (**DCh^−^**) and lithocholate (**LCh^−^**) (Figure 1), by four commercial cyclodextrin derivatives: 2-hydroxypropyl-**β-CD** (**HP-β-CD**), 2-hydroxypropyl-**γ-CD** (**HP-γ-CD**), randomly methylated **β-CD** (**RM-β-CD**), and sulfobutylether-**β-CD** sodium salt (**SB-β-CD**) with varying degrees of substitution (DS ≈ 4, 6, 10). Complexation was studied by means of ITC, which enabled complete thermodynamic characterization of the binding event, while ^1^H NMR spectroscopy provided qualitative structural insight into the complexes formed. Obtained thermodynamic parameters were compared with those of the parent **β-CD** and **γ-CD** determined under identical conditions [17], which allowed us to delineate the effect of cyclodextrin substitution on binding stoichiometry, affinity, and complex solubility with both gonane isomers and charged *cis*-bile salt anions.

This work provides the first full thermodynamic characterization of neutral steroid stereoisomers’ inclusion reactions with substituted cyclodextrins, and a systematic study of non-conjugated bile salt anion complexation by **SB-β-CD** with varying degrees of substitution (DS ≈ 4, 6, 10). Two important findings stand out: the suppression of the *trans*-steroid: (**β-CD**)_2_ complex formation upon cyclodextrin substitution and the emergence of the degree of substitution among sulfobutylether derivatives as a potential bile-salt-anion-selectivity filter.

**Table 1:** Thermodynamic parameters for the complexation of the neutral steroids and bile salt anions (Figure 1) with the parent cyclodextrins determined in the companion study [17], compared with literature values for related host–guest systems. Parameters for the substituted cyclodextrins are given under Section 2.

Guest	Host	log *K*° ^a^	Δ_r_*G*°/kJ mol^−1^	Δ_r_*H*°/kJ mol^−1^	−*T*Δ_r_*S*°/kJ mol^−1^
**E**	**β-CD**	4.72	−26.91	−36.97	10.05
	**γ-CD**	4.76	−27.2	−11.18	−16.0
**A**	**β-CD**	5.19 4.74 (1:2)	−29.62 −27.04	−33.6 −46.47	4.0 19.43
		2.37 (ref. [42], ^1^H NMR, D_2_O)			
	**γ-CD**	4.86	−27.76	−8.33	−19.4
**A2**	**β-CD**	n.d. ^b^			
	**γ-CD**	4.6	−26.1	−6.1	−20
**T**	**β-CD**	4.56 3.85 (1:2)	−26.0 −21.95	−25.6 −29.6	−0.4 7.6
		4.25 (ref. [43], phase solubility method)			
	**γ-CD**	4.51	−25.72	−7.10	−18.62
**Ch^−^**	**β-CD**	3.64	−20.8	−28.2	7.5
		3.60 (ref. [44], ITC, H_2_O)	−20.54	−27.0	6.46
		3.61 (ref. [45], ITC, phosphate buffer pH = 7.2)	−20.60	−22.98	2.38
		3.50 (ref. [46], flow microcal., H_2_O)	−20.0	−26.0	6.0
		3.90 (ref. [47], ^13^C NMR, D_2_O)			
		2.97 (ref. [48], CMC-induced shifts, H_2_O)			
	**γ-CD**	3.77	−21.50	−10.98	−10.53
		3.51 (ref. [49], ^13^C NMR, D_2_O)			
**DCh^−^**	**β-CD**	3.85	−21.99	−34.6	12.6
		3.69 (ref. [45], ITC, phosphate buffer pH = 7.2)	−21.03	−25.79	4.76
		4.79 (ref. [46], flow microcal., H_2_O) ^c^	−27.3	−21.8	−5.5
		3.57 (ref. [48], CMC-induced shifts, H_2_O)			
	**γ-CD**	4.44	−25.3	−7.3	−18.0
		3.30 (ref. [49], ^13^C NMR, D_2_O)			
**LCh^−^**	**β-CD**	6.23	−35.6	−39.9	4.3
		>6 (for **HLCh,** ref. [50], ITC, water/DMSO)			
	**γ-CD**	6.29	−35.92	−15.76	−20.2

^a^ For each guest and host pair, the first row gives the parameters from the companion study (ref. [17], determined by ITC in H_2_O (or 5 mmol dm^−3^ NaOH(aq) for **DCh^−^** and **LCh^−^**) at 298 K); all further lines are literature values with references and the corresponding methods/conditions indicated. Values labeled (1:2) refer to the successive thermodynamic parameters for 1:2 (guest:host) complex; all other companion values correspond to 1:1 complexes. ^b^ GH and GH_2_ complexes are indicated, but the data could not be reliably fitted. ^c^ **DCh^−^** also forms a 1:2 complex with **β-CD** (literature log *K*° = 2.86, ref. [46]).

## 2. Results and Discussion

### 2.1. Complexation of Neutral Steroids with Substituted Cyclodextrins

The microcalorimetric titrations allowed for detailed thermodynamic characterization of the complexation reactions, while ^1^H NMR spectroscopy provided information concerning the realized steroid–host contacts. Standard complex stability constants and reaction enthalpies were determined by non-linear regression analysis of calorimetric data, hence also rendering the Δ_r_*G*° and Δ_r_*S*° (Δ_r_*G*° = −*RT* ln *K*°; Δ_r_*G*° = Δ_r_*H*° − *T*Δ_r_*S*°). Before discussing the obtained results, it should be noted that the herein used commercial cyclodextrin derivatives contain isomers differing in substitution degree and position, so that several complexes coexist in solution. The determined thermodynamic parameters, collected in Table 2, therefore correspond to an average over the contributing species.

#### 2.1.1. 2-Hydroxypropylated Cyclodextrins

**Figure 2:**
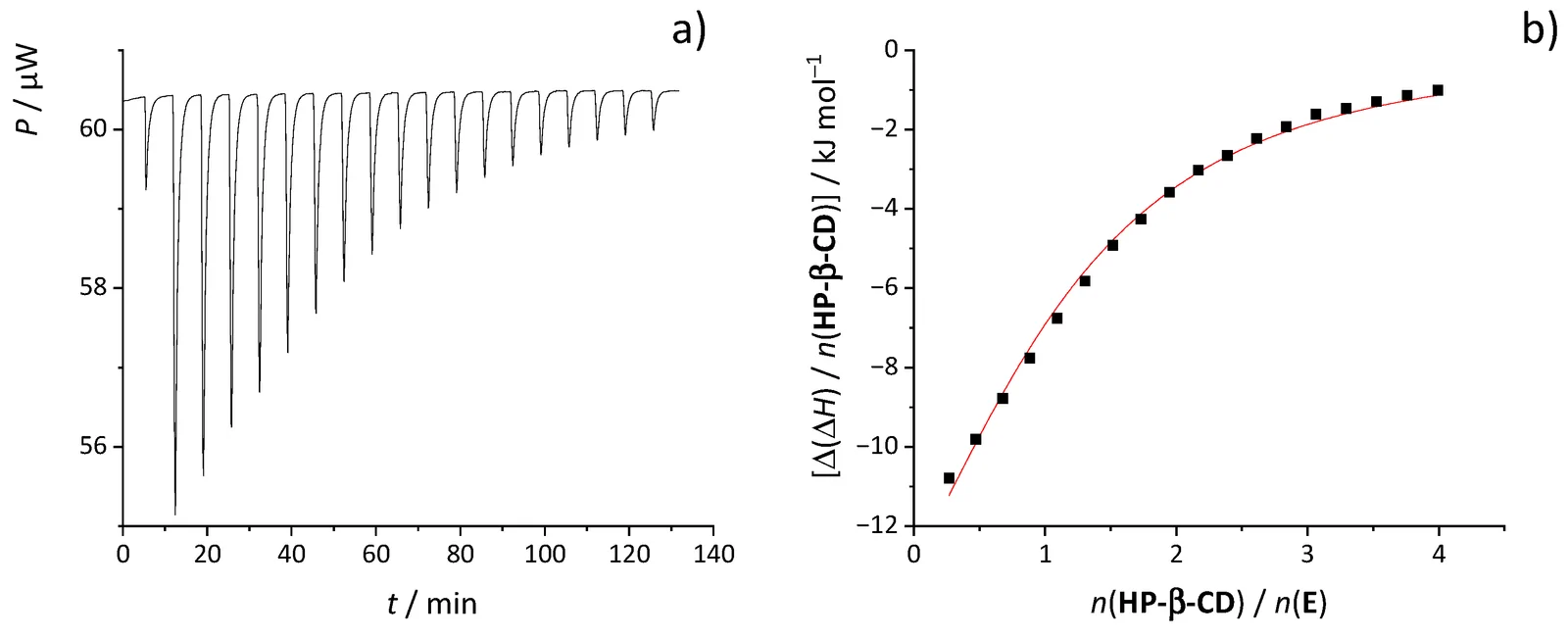
Microcalorimetric titration of **E** (*c*_0_ = 1.03 × 10^−4^ mol dm^−3^, *V* = 1.45 cm^3^) with **HP-β-CD** (*c* = 1.98 × 10^−3^ mol dm^−3^) in H_2_O at 298 K. (**a**) Thermogram. (**b**) Dependence of the normalized successive enthalpy change on the host-to-guest ratio. ■ experimental; − calculated.

The calorimetric data for **E** and **A** binding with **HP-β-CD** (Figure 2 and Figure S1) were adequately described by a 1:1 binding model, while introduction of higher-stoichiometry species did not improve the fit. This finding is in sharp contrast with the results of our previous study [17], where formation of 1:1 and 1:2 (guest:host) complexes of quasi-planar *trans-*gonane isomers with **β-CD** was clearly observable from the inflection point at approximately 2:1 host-to-guest molar ratio. On the other hand, due to steric restrictions [17], the V-shaped *cis*-isomer **E** formed exclusively 1:1 complexes with native cyclodextrin. One can hence conclude that the introduction of bulky hydroxypropyl groups prevents the coordination of a second **β-CD** molecule to *trans*-gonane isomers. Concerning the stability of **A** and **E** complexes with the substituted host, hydroxypropylation decreases receptor affinity for both stereoisomers (Δlog *K*° = −0.65 (**E**) and −0.74 (**A**), Table 1 and Table 2). The lower log *K*° values stem from notably less exothermic binding with **HP-β-CD** (Δ(Δ_r_*H*°) ≈ 10−14 kJ mol^−1^), which is partially compensated by a more favorable complexation entropy than for the parent cyclodextrin (enthalpy–entropy compensation) [51].

The considerable stability of the etiocholanolone complex with **HP-γ-CD** (Figure S2, Table 2) is almost entirely a consequence of favorable complexation entropy (−*T*Δ_r_*S*° = −21.4 kJ mol^−1^; Δ_r_*H*° = −3.3 kJ mol^–1^), while the weakly endothermic binding of **A** with this **γ-CD** derivative (Figure S3) could not be characterized calorimetrically (Δ_r_*H*° ≳ 0). Importantly, lower concentrations of the steroid in ITC titrations of **A** (compared to **E**) were used, due to its significantly lower aqueous solubility. Increasing the host concentration would produce even larger heat effects from titrant dilution, still leaving the corrected data within the noise range. However, to test whether complexation occurs, ^1^H NMR spectra of **A** and its mixture with **HP-γ-CD** in D_2_O were recorded (Figure S5), and the expectedly smaller changes in chemical shifts of the guest methyl protons (compared to the **HP-β-CD** analog) were interpreted as weak or shallow binding. It should be mentioned that due to large negative Δ_r_*C_p_*°, characteristic of cyclodextrin inclusion reactions with steroids and bulky cyclic aliphatic guests [17,35,52,53,54], the binding thermodynamics is expected to shift from predominantly or even completely entropy-driven (classical hydrophobic effect) at lower temperatures towards enthalpy-dominated (non-classical hydrophobic effect) as temperature increases.

Three important conclusions can be drawn from the comparative studies of native and 2-hydroxypropylated receptors: First, the difference in the complexation parameters for reactions with seven- and eight-membered derivatives mirrors that between the parent **β-CD** and **γ-CD**; i.e., irrespective of substitution, the cavity water of the larger receptor is more extensively hydrogen-bonded, and its expulsion is therefore less exothermic [17,40]. Second, the calorimetric data for native and 2-hydroxypropylated hosts (Table 1 and Table 2) reveal that rim substitution reduces the affinity of the seven-membered receptor more strongly, plausibly because the narrower, more rigid rim of **β-CD** allows a closer contact between the appended chains and the guest. Third, the differences in Δ_r_*H*° and Δ_r_*S*° values for complexation with substituted and parent cyclodextrins indicate that the functionalization of receptors pushes the complexation thermodynamics towards the classical hydrophobic effect (entropically more favorable binding). This can be rationalized by the more extensive dehydration of the presumably classically hydrated steroid backbone and introduced hydroxypropyl groups, which is consistent with endothermic complexation of androsterone by **HP-γ-CD** and the literature data on the hydroxypropyl group hydration [34,35,55].

Turning attention to the complex structure, the ^1^H NMR spectra of the host–guest mixtures (Figure S4 and Figure S5) confirm the inclusion of both isomers. Specifically, the methyl-proton signals of **A** and **E** experience a downfield shift upon cyclodextrin addition, indicating their incorporation within the receptor cavities. For **A**, both methyl signals shift by a similar amount, indicating comparable inclusion depth and similar chemical environment, whereas for the *cis*-isomer **E**, the two shifts differ appreciably (particularly in the case of **HP-β-CD**), pointing to a different complex structure (i.e., peripheral inclusion of one of the methyl groups), which is to be expected considering the difference in **A** and **E** geometry. As for the parent receptors [17], the shifts induced by the **γ-CD** derivatives (Figure S5) are again markedly smaller than those induced by the **β-CD** derivatives.

#### 2.1.2. Randomly Methylated β-Cyclodextrin

**Figure 3:**
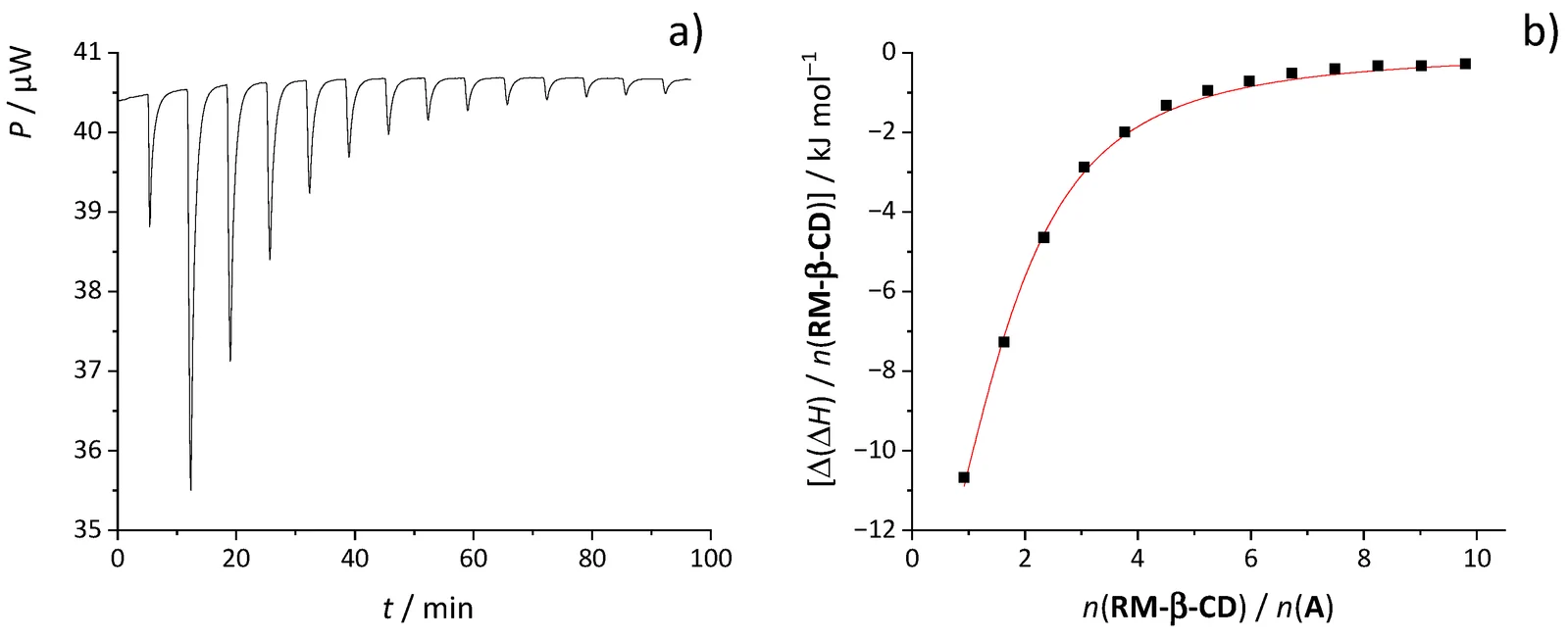
Microcalorimetric titration of **A** (*c*_0_ = 3.06 × 10^−5^ mol dm^−3^, *V* = 1.45 cm^3^) with **RM-β-CD** (*c* = 2.00 × 10^−3^ mol dm^−3^) in H_2_O at 298 K. (**a**) Thermogram. (**b**) Dependence of the normalized successive enthalpy change on the host-to-guest ratio. ■ experimental; − calculated.

The methyl groups of **RM-β-CD** extend the rim less than the hydroxypropyl chains, resulting in a shorter hydrophobic cavity [34,36,56]. Nevertheless, this **β-CD** derivative, like the **HP-β-CD**, forms only a 1:1 inclusion complex with androsterone (Figure 3), suggesting that even the slightest extension of the cavity hinders the attachment of the second receptor molecule to the quasi-planar backbone of *trans*-steroids. This is most likely due to steric incompatibility of the modified **β-CD** and the *trans*-gonane backbone; however, the contacts realized between the OH groups of native receptors in a 1:2 complex could also lead to additional stabilization of the product [17,36].

That said, the substitution has only a minor effect on the receptor affinity for **E** (Figure S6) and **A** (comparison with the **A**:**β-CD** complex, Table 1 and Table 2). As for **HP-β-CD**, the inclusion of both isomers into **RM-β-CD** is characterized by less exothermic and more entropy-driven complexation than for the parent **β-CD**. However, the attachment of methyl groups affects the binding affinity less than hydroxypropylation despite the substantially higher degree of substitution of the randomly methylated derivative (DS ≈ 12 vs. 5.6).

**Figure 4:**
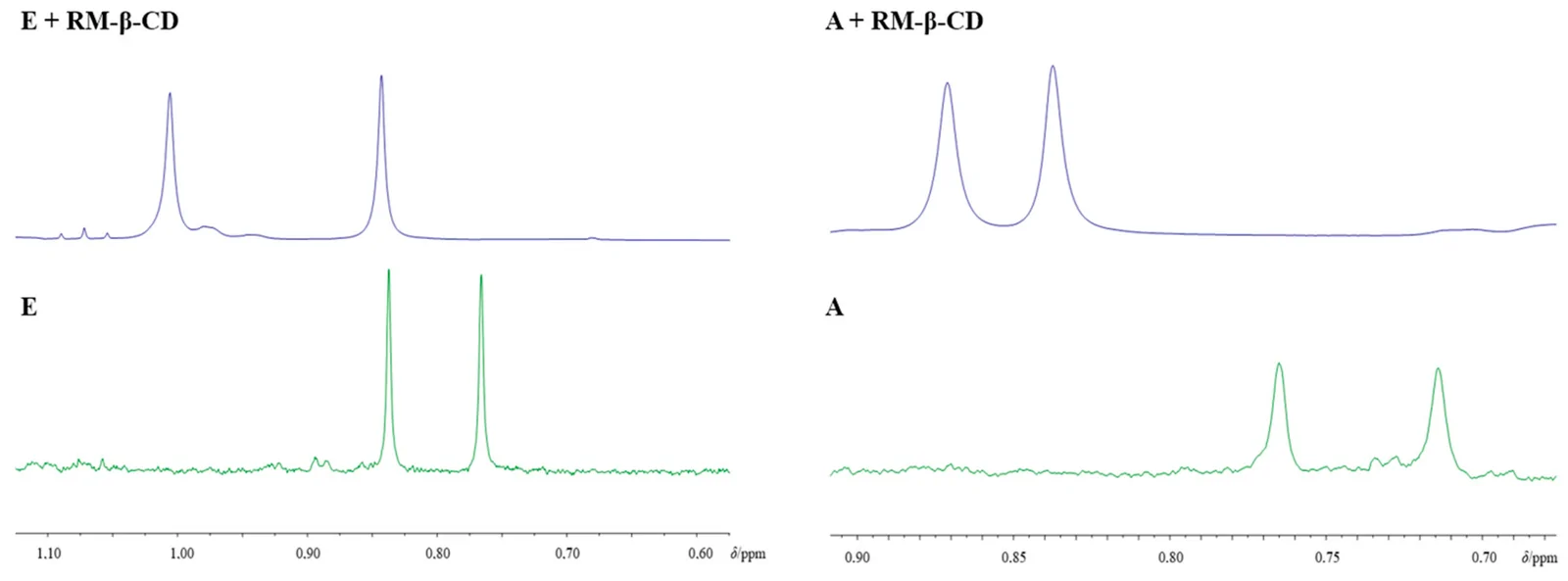
Selected regions of the ^1^H NMR spectra of solutions of **E** and **A** and of their mixtures with **RM-β-CD** in D_2_O at 298 K.

The ^1^H NMR spectra (Figure 4) reveal that both methyl signals of **A** shift by about 0.1 ppm, indicating complete inclusion of rings B and C. Conversely, the analogous signals of **E** experience different changes upon complexation with **RM-β-CD** (Δ*δ* ≈ 0.15 and 0.07 ppm, Figure 4), which can, as in the case of **HP-β-CD**, be explained by more shallow incorporation of one of the guest’s methyl groups into the cyclodextrin cavity.

#### 2.1.3. Sulfobutylether-β-Cyclodextrin

**Figure 5:**
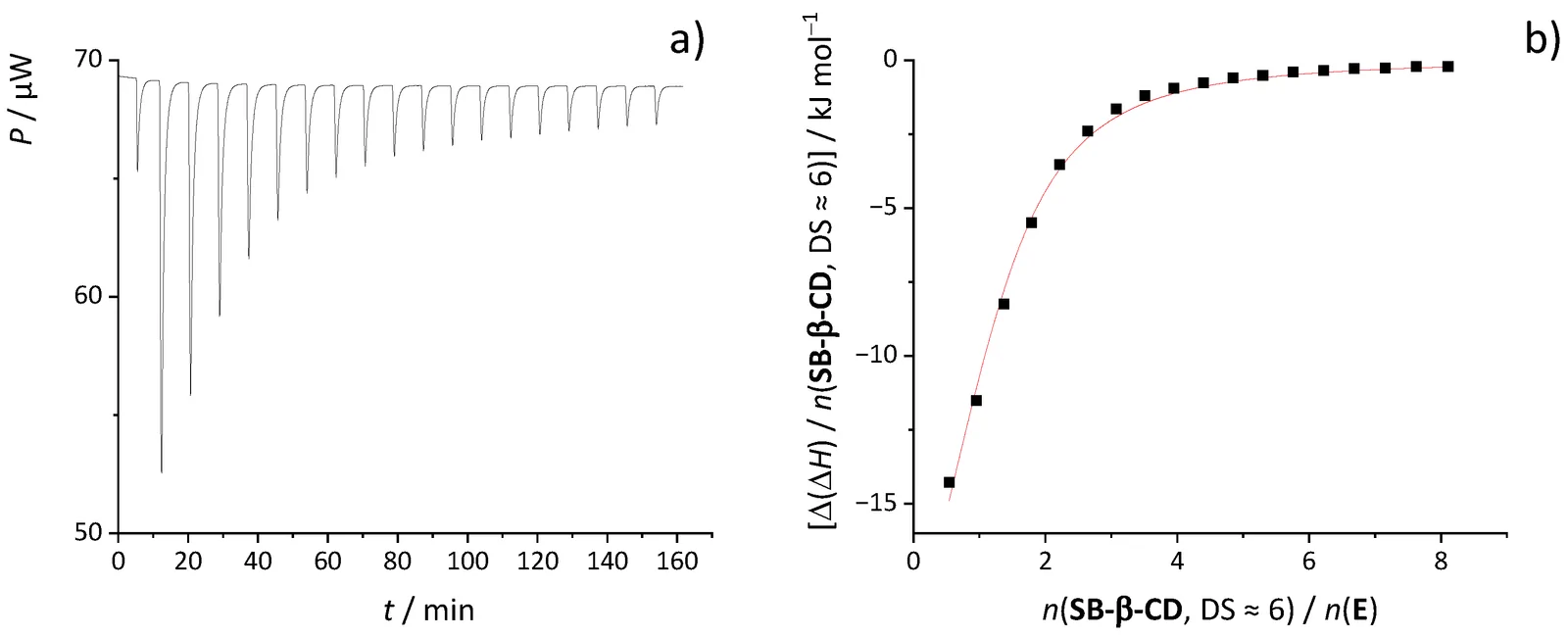
Microcalorimetric titration of **E** (*c*_0_ = 1.03 × 10^−4^ mol dm^−3^, *V* = 1.45 cm^3^) with **SB-β-CD** (DS ≈ 6, *c* = 9.98 × 10^−3^ mol dm^−3^) in H_2_O at 298 K. (**a**) Thermogram. (**b**) Dependence of the normalized successive enthalpy change on the host-to-guest ratio. ■ experimental; − calculated.

Although **SB-β-CD** (DS ≈ 6) bears longer, negatively charged sulfobutylether substituents, the modification affects binding thermodynamics less than in the case of **HP-β-CD** with a comparable degree of substitution (DS ≈ 5.6) (Table 2, Figure 5 and Figure S7). This likely reflects weaker interactions of the butyl chains and the herein studied steroids, resulting in less extensive dehydration of the extended cyclodextrin cavity and the gonane backbone compared to conjugated bile salt anions, where additional interactions with CD substituents result in more pronounced dehydration [38,40]. Accordingly, the shift toward entropically more (and enthalpically less) favorable binding relative to the parent **β-CD** is not as prominent as for **HP-β-CD**. As could be expected, sulfobutylether-**β-CD** formed exclusively 1:1 complexes with both gonane isomers.

As in the case of hydroxypropylated and methylated receptors, the change in chemical shift of both androsterone CH_3_ group signals in ^1^H NMR spectra upon complexation with **SB-β-CD** is similar (Δ*δ* ≈ 0.17 ppm), whereas for **E**, these changes differ significantly and amount to Δ*δ* ≈ 0.2 and 0.1 ppm (Figure S8).

Importantly, we note that, unlike native **β-CD**, all of the investigated derivatives form soluble complexes with **A** (Figure S9), indicating their potential use as solubilizers of *trans*-gonane isomers. It should be noted that phase-solubility measurements, which should be performed to determine the solubilization effect quantitatively, are underway.

### 2.2. Complexation of Bile Salts with Substituted Cyclodextrins

**Figure 6:**
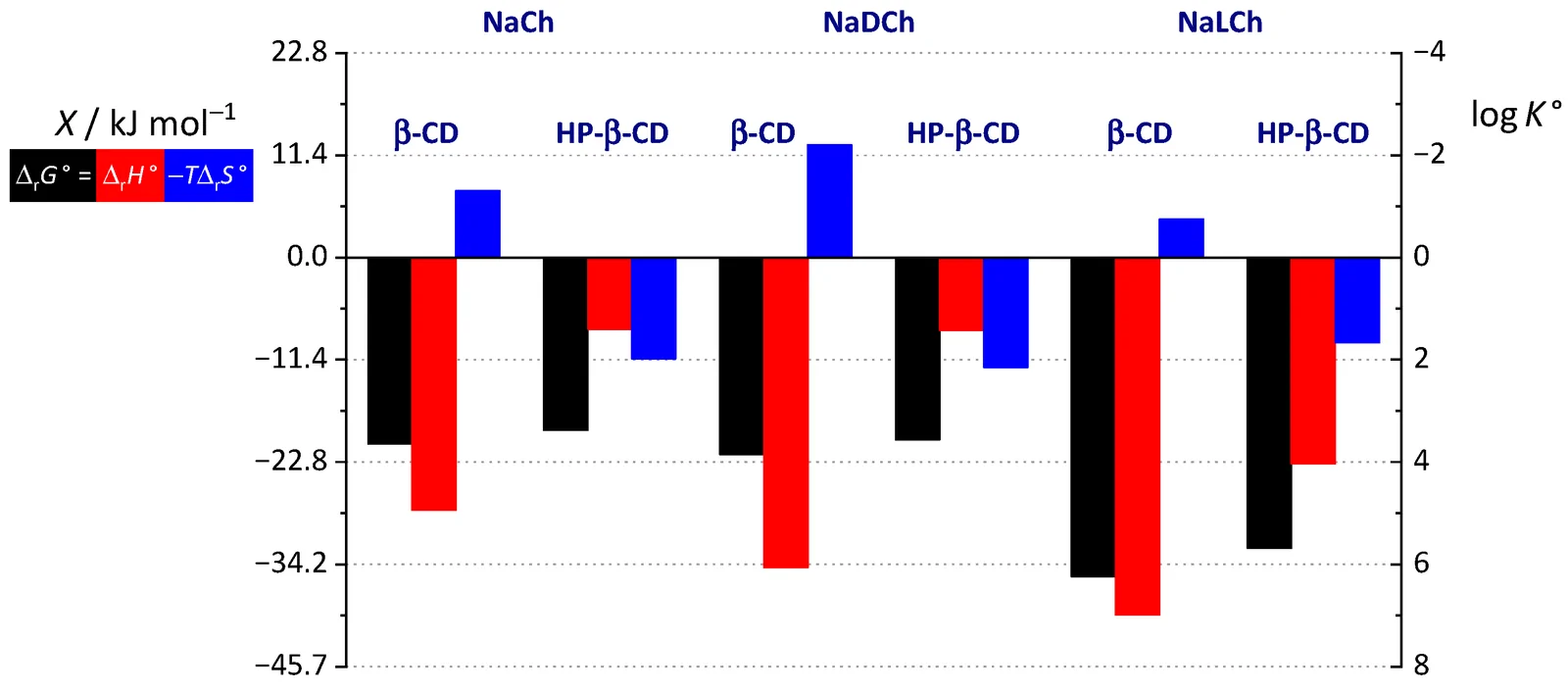
Thermodynamic reaction parameters for the complexation of **NaCh**, **NaDCh**, and **NaLCh** with **β-CD** (taken from ref. [17]) and with **HP-β-CD** in H_2_O for **NaCh** and 5 mmol dm^−3^ NaOH(aq) for **NaDCh** and **NaLCh** at 298 K.

**Figure 7:**
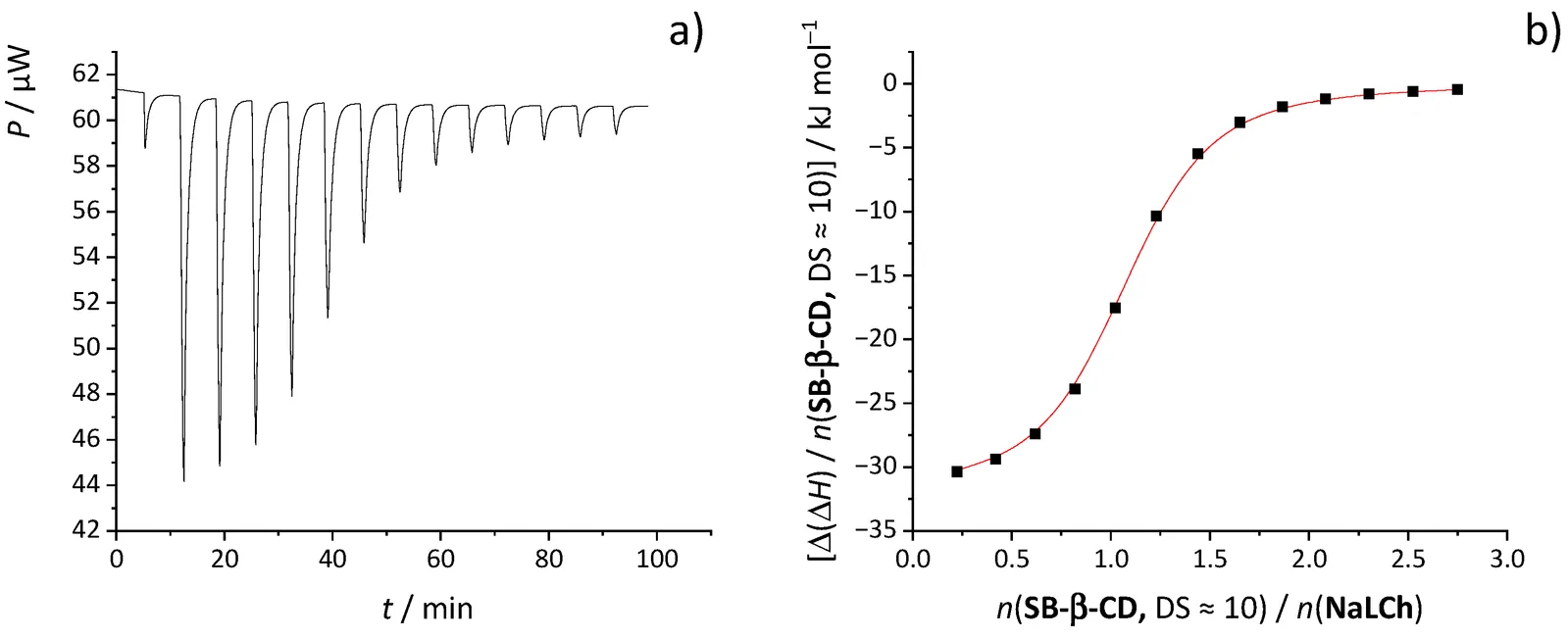
Microcalorimetric titration of **NaLCh** (*c*_0_ = 1.22 × 10^−4^ mol dm^−3^, *V* = 1.45 cm^3^) with **SB-β-CD** (DS ≈ 10, *c* = 1.95 × 10^−3^ mol dm^−3^) in 5 mmol dm^−3^ NaOH(aq) at 298 K. (**a**) Thermogram. (**b**) Dependence of the normalized successive enthalpy change on the host-to-guest ratio. ■ experimental; − calculated.

Considering that the affinity of **β-CD** and **γ-CD** for bile salt anions decreases with increasing hydroxylation of the gonane backbone [41,57], and in line with earlier work on synthetic cyclodextrin hosts [29,31,34,35,38,39], we examined the complexation of cholate (**Ch^−^**), deoxycholate (**DCh^−^**), and lithocholate (**LCh^−^**) with the hydroxypropylated (**HP-β-CD** and **HP-γ-CD**) and sulfobutylether cyclodextrin derivatives (Figure 6, Figure 7 and Figure S10–S20, Table 3). The influence of the substitution degree was analyzed in detail for the two boundary guests: lithocholate, bearing a single OH group at C3, and the most polar cholate, with two additional OH groups at C7 and C12 (Figure 1).

As already found for the neutral [17] and charged steroids [34,58], hydroxypropylation lowers the **β-CD** affinity only slightly for **Ch^−^** and **DCh^−^**, while the decrease is larger for the most hydrophobic **LCh^−^** (Figures S10–S14, Table 3). The data presented in Figure 6 suggest partial enthalpy–entropy compensation. In this respect, it should be noted that the reaction entropy is derived from the experimentally determined reaction Gibbs energy and enthalpy, and these values are therefore necessarily correlated, which may affect the observed compensation. However, the pronounced differences in Δ_r_*H*° and −*T*Δ_r_*S*° observed for all three bile anions (particularly **Ch^−^** and **DCh^−^**) with **β-CD** and **HP-β-CD** indicate that hydroxypropylation indeed leads to partial enthalpy–entropy compensation. This substitution effect on complexation with seven-membered cyclodextrins can be rationalized by the more extensive dehydration of both the classically hydrated gonane backbone and the hydroxypropyl groups [35,40,59]. Notably, hydroxypropylation affects the complexation enthalpy and entropy more strongly for bile anions than for neutral steroids (Δ(Δ_r_*H*°) up to 13.97 (**E**) and 26.49 (**DCh**^−^) kJ mol^−1^; −*T*Δ(Δ_r_*S*°) up to −10.25 **(E**) and −24.85 (**DCh**^−^) kJ mol^−1^, Table 2 and Table 3), despite having a smaller effect on complex stability (Δlog *K*° up to −0.74 (**A**) vs. −0.55 (**LCh^−^**)). This suggests that the classically hydrated alkyl side chain at the D ring of bile salt anions also contributes to the complexation process.

For the larger **HP-γ-CD**, inclusion of **Ch**^−^ (Figure S11) and **DCh**^−^ (Figure S13) becomes almost isoenthalpic and can no longer be characterized by ITC. Together with earlier reports [34,35,40], these results show that hydroxypropylation of both seven- and eight-membered cyclodextrins pushes the thermodynamic fingerprint toward the classical hydrophobic effect, irrespective of the charge or the degree of hydroxylation of the steroid.

Because both the bile salt anions and the sulfobutylether groups carry a negative charge, the substitution degree might be expected to affect complex stability more strongly than for the hydroxypropylated hosts. As noted in the Introduction, however, Holm et al. found comparable affinities of **SB-β-CD** (DS ≈ 7) and native **β-CD** toward tauro- and glycoconjugated bile salts and their deoxy analogues [38]. To examine how substitution degree and the number of steroidal hydroxyl groups govern recognition, we studied **SB-β-CD**s of DS ≈ 4, 6, and 10 with cholate and lithocholate, the most polar and the most hydrophobic of the three anions.

The parameters in Table 3 show that all three **SB-β-CD**s bind the anions less strongly than the parent **β-CD**. For cholate, stability decreases with increasing DS. The decrease is moderate between DS ≈ 4 and 6 but becomes marked at DS ≈ 10, which is consistent with its lower hydrophobicity. The three hydroxyl groups most likely hinder its deep penetration into the cavity; however, this reasonable assumption requires further confirmation by means of ROESY NMR. For lithocholate, log *K*° falls smoothly with DS, and the three hosts give very similar Δ_r_*H*° and Δ_r_*S*°, indicating that the guest does not interact simultaneously with all sulfobutyl ether chains. Cholate is one of the two most abundant bile acids in the human bile acid pool [60]. The presented results hence indicate that its competition with an active pharmaceutical ingredient for the cavity of seven-membered cyclodextrins can be reduced by choosing highly substituted **SB-β-CD**s as hosts.

Interestingly, the introduction of sulfobutylether groups raises the host selectivity for anions with fewer OH groups on rings B and C, contrary to the loss of selectivity usually reported upon CD substitution [29,34,40].

### 2.3. Comparison with Literature Data

The stability constants obtained here and in the companion study lie within the scattered literature range for parent cyclodextrins (Table 1), where most values come from NMR titrations and, for bile salt anions, vary appreciably with method and conditions. Calorimetric data for substituted-cyclodextrin complexes of neutral stereoisomers and of non-conjugated bile salts are, to our knowledge, scarce: the only reported study of non-conjugated bile salts with substituted **CD**s [58] neither specifies the substitution degree nor uses unbuffered water (TRIS-NaCl instead), so the tauro- and glyco-conjugated bile salts remain the closest documented systems, studied with hydroxypropylated and methylated cyclodextrins [34,36,40]. As already mentioned, these exhibit the same qualitative dependence of Δ_r_*H*° and Δ_r_*S*° on the substitution degree found here; in fact, the increments estimated by Schönbeck et al. [34], about 2.2–3.6 kJ mol^−1^ added to Δ_r_*H*° and 1.7–3.6 kJ mol^−1^ removed from −*T*Δ_r_*S*° per hydroxypropyl group, agree well with the differences between our native and 2-hydroxypropylated hosts (Table 1, Table 2 and Table 3). The agreement is better for bile salt anions than for neutral steroids, as expected from their stronger interaction with the rims. The present work thus reproduces and extends the framework of Schönbeck, Holm and colleagues [34,35,36,38,40,41], established for conjugated bile salts, to non-conjugated analogues and neutral steroid stereoisomers. The substitution-induced shift of Δ_r_*H*° and Δ_r_*S*° holds for both *trans-* and *cis*-steroid isomers and does not depend on the position of polar guest groups, which differ across the series: C3 in all guests, additionally C7 and C12 in cholate and deoxycholate, and C24 in the conjugated bile acids studied in the literature (Figure 1).

## 3. Materials and Methods

### 3.1. Materials

Etiocholanolone (**E**, Sigma-Aldrich, Steinheim, Germany, ≥98.0%), androsterone (**A**, Sigma-Aldrich, Steinheim, Germany, ≥98%) and sodium cholate (**NaCh**, Fluka, Buchs, Switzerland, ≈98%) were used as received. The macrocyclic receptors β-cyclodextrin (**β-CD**, Sigma-Aldrich, Steinheim, Germany, ≥98%), 2-hydroxypropyl-β-cyclodextrin (**HP-β-CD**, DS ≈ 5.6, Sigma-Aldrich, ≥98%; Batch no. 332607-1004), 2-hydroxypropyl-γ-cyclodextrin (**HP-γ-CD**, DS = 4.0–5.6, Ashland, Wilmington, DE, USA, >99.0%; Batch no. A1306A0113), randomly methylated β-cyclodextrin (**RM-β-CD**, DS ≈ 12, CycloLab, Budapest, Hungary, >95%; Batch no. CYL-2738), and sulfobutylether-β-cyclodextrin sodium salt (**SB-β-CD**, DS ≈ 4, 6 and 10, all CycloLab, Budapest, Hungary, >95%; Batch no. CYL-4220 (DS ≈ 4); CYL-3561 (DS ≈ 6); CYL-3321 (DS ≈ 10)) are commercial mixtures with the nominal average degree of substitution (DS) indicated per cyclodextrin molecule (Figure 1); all receptors were dried at 150 °C for 3 h prior to use. Sodium deoxycholate (**NaDCh**) and sodium lithocholate (**NaLCh**) were prepared from deoxycholic acid (**HDCh**, Sigma-Aldrich, Steinheim, Germany, ≥99.0%) and lithocholic acid (**HLCh**, Sigma-Aldrich, Steinheim, Germany, ≥95%), and sodium hydroxide (NaOH, Gram-Mol, Zagreb, Croatia, *pro analysi*) was used for the deprotonation of the bile acids. Milli-Q water was used as the solvent for all microcalorimetric experiments, except for the titrations involving **NaDCh** and **NaLCh**, which were carried out in 5 mmol dm^−3^ NaOH(aq) owing to anion hydrolysis and the poor aqueous solubility of **HDCh** and **HLCh** in H_2_O, and deuterium oxide (Sigma-Aldrich, Steinheim, Germany, >99.9% D) for the NMR experiments.

The influence of pH and ionic strength was ruled out in our previous studies with native cyclodextrins, where virtually the same values of all thermodynamic parameters were obtained in water and in 5 mmol dm^−3^ NaOH(aq) for **NaDCh** at lower concentration with **β-CD** (Table S1).

### 3.2. Isothermal Titration Calorimetry

ITC measurements were performed with a Malvern MicroCal VP-ITC calorimeter (Malvern Panalytical, Malvern, UK, *V*_cell_ = 1.45 cm^3^). Titrations were carried out by stepwise addition of the host (titrant) solution to a solution of the guest (titrand). The concentration and volume of the titrant were varied according to the concentration of the titrand. Constant stirring (300 rpm) was applied, and the time between additions was 350–500 s. Blank experiments were carried out for each titration, and the heats of titrant dilution were subtracted from those measured in the titration. Microcal OriginPro 7.0 (OriginLab Corporation, Northampton, MA, USA), supplied by the manufacturer, was used for data acquisition and processing. The complex stoichiometry was fixed at *N* = 1 (one set of sites model). Further, the sigmoidal shape of the titration curves in the case of tighter binding (log *K*° above 4), with the inflection point at a host-to-guest molar ratio of approximately 1, clearly indicated 1:1 stoichiometry of the formed complexes. Fitting the data with a sequential two-site model, which accounts for a higher-stoichiometry complex, did not improve agreement between fitted and experimental values; therefore, the simplest 1:1 model was retained. All titrations were conducted at least in triplicate, and the parameters are reported as means with the standard errors of the mean as a measure of uncertainty.

Due to low solubility of **A** (compared to **E**), microcalorimetric titrations could not be conducted at higher initial guest concentrations.

### 3.3. ^1^H NMR Spectroscopy

NMR experiments were performed in D_2_O at 298 K on a Bruker Avance III HD 400 MHz/54 mm spectrometer (Bruker BioSpin GmbH, Rheinstetten, Germany) equipped with an inverse broadband room-temperature probe (5 mm PA BBI 1H/D–BB). One-dimensional ^1^H spectra of the guests, hosts, and their mixtures were recorded with standard Bruker pulse sequences and processed with TopSpin software (v. 3.6.2) supplied by the manufacturer.

## 4. Conclusions

Microcalorimetric and ^1^H NMR measurements show that functionalization of the cyclodextrin rim modulates both the stability and the driving force of steroid and bile salt inclusion in a systematic way. Substitution lowers the complex stability and shifts the thermodynamic signature towards less exothermic, entropically more favorable binding, i.e., it induces a partial enthalpy–entropy compensation. The effect is largest for the hydroxypropylated hosts.

Unlike the parent **β-CD**, all its derivatives form exclusively 1:1 complexes with the studied steroids, irrespective of gonane isomerism. Rim substitution therefore suppresses the attachment of a second host molecule to the quasi-planar *trans*-isomer, removing the isomerism-controlled difference in stoichiometry found for the native host in the companion study [17].

For the anionic guests, the affinity of the sulfobutylether host for the most polar cholate anion decreases steeply once the degree of substitution exceeds about six, which we attribute to electrostatic repulsion between the peripheral sulfonate groups and the guest carboxylate. However, this effect is significantly less pronounced for lithocholate, which is expected to reside deeper inside the cavity, thereby being less affected by rim substitution. This finding suggests that not only the conjugation of the bile salts [38,39] but also the number of OH groups on the steroid backbone significantly influences the substituted CD affinity for these guests. Accordingly, both the host substitution degree and the number and type of polar groups attached to the gonane backbone could be used as a lever for fine-tuning the recognition of charged steroids. Taken together, the herein presented results provide a basis for choosing cyclodextrin excipients that resist cholate anion competition with active pharmaceutical ingredients, generally have fewer contraindications and do not form sparingly soluble complexes with *trans*-steroids like the native **β-CD** does (Figure S9). These results open new paths for investigating the influence of gonane isomerism on binding stoichiometry and thermodynamics with both parent and substituted cyclodextrins, e.g., by studying the complexation of *trans*-bile salt derivatives (*allo*-bile salts).

Regarding the full structural rationalization of the observed substitution effects, further studies are underway. As in our previous studies of hydrophobically driven inclusion, these will combine computational and spectroscopic (ROESY NMR) investigations of the reactants and product to elucidate the complex structural features, alongside DOSY NMR experiments to confirm the binding stoichiometry and equilibrium between the free and bound reactants.

## Figures and Tables

**Table 2:** Thermodynamic parameters of the complexation of etiocholanolone (**E**) and androsterone (**A**) with substituted cyclodextrins in H_2_O at 298 K ^a^.

Guest	Host	log *K*°	Δ_r_*G*°/kJ mol^−1^	Δ_r_*H*°/kJ mol^−1^	−*T*Δ_r_*S*°/kJ mol^−1^
**E**	**HP-β-CD**	4.07(3)	−23.2(2)	−23.0(4)	−0.2(5)
	**RM-β-CD**	4.42(1)	−25.21(4)	−29.0(3)	3.8(4)
	**SB-β-CD** (DS ≈ 6)	4.3(4)	−24(2)	−26(1)	2(1)
	**HP-γ-CD**	4.3(1)	−24.7(6)	−3.3(3)	−21.4(9)
**A**	**HP-β-CD**	4.45(6)	−25.4(3)	−22.82(6)	−2.59(7)
	**RM-β-CD**	4.54(1)	−25.9(5)	−29.7(3)	3.8(4)
	**SB-β-CD** (DS ≈ 6)	4.63(2)	−26.4(1)	−28.2(2)	1.7(2)
	**HP-γ-CD**	− ^b^	− ^b^	≳0 ^b^	− ^b^

^a^ Values refer to the process G + H ⇄ GH; the standard error of the mean (*N* = 3–5) is given in parentheses as the uncertainty of the last significant digit. ^b^ Binding observed by NMR but not quantifiable by ITC (Δ_r_*H*° ≳ 0).

**Table 3:** Thermodynamic parameters of the complexation of bile salt anions with substituted cyclodextrins in H_2_O for **NaCh** and 5 mmol dm^−3^ NaOH(aq) for **NaDCh** and **NaLCh** at 298 K compared with literature parameters for related host–guest systems ^a^.

Guest	Host	log *K*°	Δ_r_*G*°/kJ mol^−1^	Δ_r_*H*°/kJ mol^−1^	−*T*Δ_r_*S*°/kJ mol^−1^
**Ch^−^**	**HP-β-CD**	3.38(1)	−19.27(5)	−8.0(1)	−11.3(2)
	**HP-β-CD** (DS not specified, ref. [58], ITC, TRIS/NaCl pH = 7.4)	3.40	−19.4	−7.9	−11.5
	**SB-β-CD** (DS ≈ 4)	3.27(4)	−18.7(2)	−19.6(3)	1.1(4)
	**SB-β-CD** (DS ≈ 6)	2.63(3)	−15.0(2)	−22.6(6)	7.6(8)
	**SB-β-CD** (DS ≈ 10)	<2 ^b^	− ^b^	− ^b^	− ^b^
	**HP-γ-CD**	− ^c^	− ^c^	≈0 ^c^	− ^c^
**DCh^−^** ^d^	**HP-β-CD**	3.57(1)	−20.36(5)	−8.11(2)	−12.25(3)
	**HP-β-CD** (DS not specified, ref. [58], ITC, TRIS/NaCl pH = 7.4)	3.65	−20.8	−10.65	−10.2
	**SB-β-CD** (DS ≈ 6)	3.07(1)	−17.52(5)	−19.0(8)	1.5(9)
	**HP-γ-CD**	− ^c^	− ^c^	≳0 ^c^	− ^c^
**LCh^−^** ^d^	**HP-β-CD**	5.68(1)	−32.44(8)	−23.0(1)	−9.5(2)
	**SB-β-CD** (DS ≈ 4)	5.98(2)	−34.1(1)	−33.0(4)	−1.1(4)
	**SB-β-CD** (DS ≈ 6)	5.60(1)	−31.95(3)	−32.26(8)	0.3(1)
	**SB-β-CD** (DS ≈ 10)	5.31(1)	−30.32(5)	−31.77(6)	1.4(1)

^a^ Values refer to the process G + H ⇄ GH; the standard error of the mean (*N* = 3–5) is given in parentheses as the uncertainty of the last significant digit. ^b^ Could not be characterized microcalorimetrically under the conditions used due to very high dilution heats. ^c^ Binding observed by NMR but not quantifiable by ITC (approximately isoenthalpic process). ^d^ Experiments performed in 5 mmol dm^−3^ NaOH(aq) owing to hydrolysis and poor aqueous solubility of **HDCh** and **HLCh**. The influence of pH and ionic strength was ruled out in our previous studies with native cyclodextrins, where virtually the same values of all thermodynamic parameters were obtained in water and in 5 mmol dm^−3^ NaOH(aq) for **NaDCh** at lower concentration with **β-CD** (Table S1).

## Data Availability

The data presented in this study are available in the article and Supplementary Materials.

## Supplementary Materials

The following supporting information can be downloaded at: https://www.mdpi.com/article/10.3390/molecules31172999/s1, Figure S1: Microcalorimetric titration of **A** with **HP-β-CD**; Figures S2 and S3: Microcalorimetric titrations of **E** and **A** with **HP-γ-CD**; Figures S4 and S5: ^1^H NMR spectra of solutions of **E** and **A** and of their mixtures with **HP-β-CD** and **HP-γ-CD**; Figure S6: Microcalorimetric titration of **E** with **RM-β-CD**; Figure S7: Microcalorimetric titration of **A** with **SB-β-CD** (DS ≈ 6); Figure S8: ^1^H NMR spectra of solutions of **E** and **A** and of their mixtures with **SB-β-CD** (DS ≈ 6); Figure S9: ^1^H NMR spectra of suspension of **A** and its mixtures with 10 equivalents of **β-CD**, **HP-β-CD**, **RM-β-CD**, and **SB-β-CD** (DS ≈ 6); Figures S10–S14: Microcalorimetric titrations of **NaCh**, **NaDCh**, and **NaLCh** with **HP-β-CD** and **HP-γ-CD**; Figure S15: ^1^H NMR spectra of solutions of **NaCh** and **NaDCh** and of their mixtures with **HP-β-CD**; Figures S16–S20: Microcalorimetric titrations of **NaCh**, **NaDCh** and **NaLCh** with **SB-β-CD** (DS ≈ 4 and 6); Table S1: Thermodynamic parameters of the complexation of **NaDCh** with **β-CD** in H_2_O and 5 mmol dm^−3^ NaOH(aq).

## Abbreviations

The following abbreviations are used in this manuscript:

**β-CD**	β-Cyclodextrin
**γ-CD**	γ-Cyclodextrin
**HP-β-CD**	2-Hydroxypropyl-β-cyclodextrin
**HP-γ-CD**	2-Hydroxypropyl-γ-cyclodextrin
**RM-β-CD**	Randomly methylated β-cyclodextrin
**SB-β-CD**	Sulfobutylether-β-cyclodextrin sodium salt
**A**	Androsterone
**A2**	Androstanedione
**E**	Etiocholanolone
**T**	Testosterone
**NaCh**	Sodium cholate
**Ch^−^**	Cholate anion
**NaDCh**	Sodium deoxycholate
**DCh^−^**	Deoxycholate anion
**NaLCh**	Sodium lithocholate
**LCh^−^**	Lithocholate anion
**HDCh**	Deoxycholic acid
**HLCh**	Lithocholic acid
DS	Degree of substitution (number of substituted OH groups per cyclodextrin molecule)
ITC	Isothermal titration calorimetry
NMR	Nuclear magnetic resonance (spectroscopy)
