# Supplementary material for: Molecular Recognition of Steroids and Bile Salts by Substituted Cyclodextrins: The Influence of Gonane Isomerism and Rim Functionalization on Complexation Thermodynamics
Source: Molecules. 2026 Aug 27;31(17):2999. doi: 10.3390/molecules31172999 (PMC13567701; doi:10.3390/molecules31172999)
Supplement: Supplementary file 1 [file molecules-31-02999-s001.zip › molecules-4524310-supplementary.pdf]

Molecular Recognition of Steroids and Bile Salts by Substituted Cyclodextrins: The Influence of Gonane Isomerism and Rim Functionalization on Complexation Thermodynamics

Andrea Usenik *, Stella Katovčić and Josip Požar *

Department of Chemistry, University of Zagreb Faculty of Science, Horvatovac 102A, HR-10000 Zagreb, Croatia; skatovcic.chem@pmf.hr

* Correspondence: ausenik.chem@pmf.hr (A.U.); pozar@chem.pmf.hr (J.P.)

SUPPORTING MATERIAL

S1. Complexation of neutral steroids with substituted cyclodextrins

S1.1. 2-Hydroxypropylated cyclodextrins

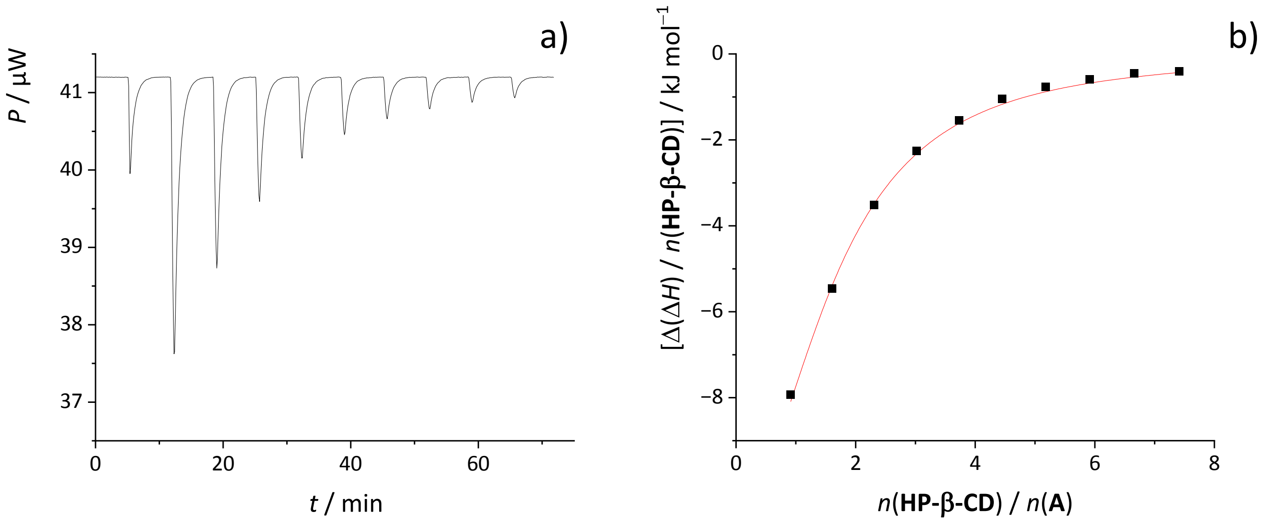

Figure S1. Microcalorimetric titration of **A** (c_0 = 3.06 × 10^{-5} mol dm^{-3}, V = 1.45 cm^3) with **HP-β-CD** (c = 1.98 × 10^{-3} mol dm^{-3}) in H_2O at 298 K. (a) Thermogram. (b) Dependence of the normalized successive enthalpy change on the host-to-guest ratio. ■ experimental; – calculated.

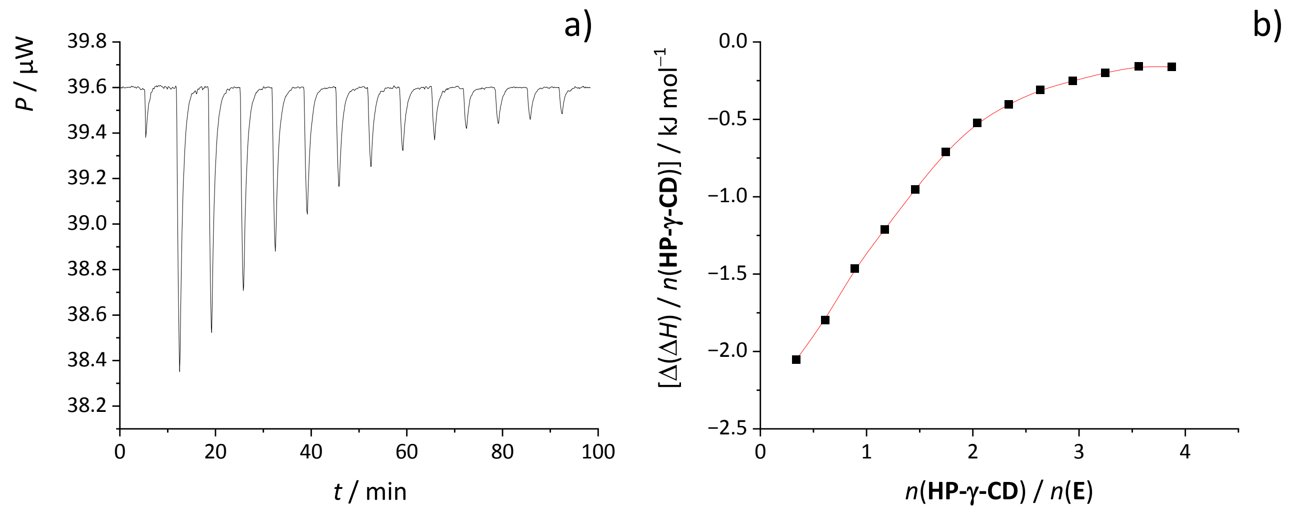

Figure S2. Microcalorimetric titration of **E** (c_0 = 1.03 × 10^{-4} mol dm^{-3}, V = 1.45 cm^3) with **HP-γ-CD** (c = 2.00 × 10^{-3} mol dm^{-3}) in H_2O at 298 K. (a) Thermogram. (b) Dependence of the normalized successive enthalpy change on the host-to-guest ratio. ■ experimental; – calculated.

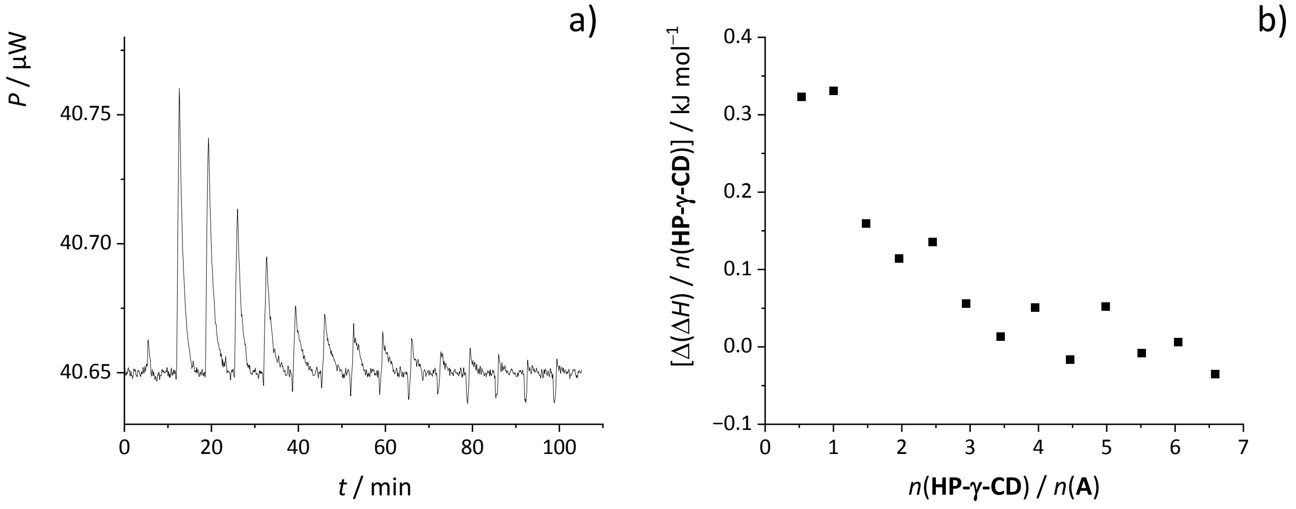

Figure S3. Microcalorimetric titration of **A** ($c_0 = 3.06 \times 10^{-5}$ mol dm^{-3}, $V = 1.45$ cm^3) with **HP-γ-CD** ($c = 9.98 \times 10^{-4}$ mol dm^{-3}) in H_2O at 298 K. (a) Thermogram. (b) Dependence of the normalized successive enthalpy change on the host-to-guest ratio.

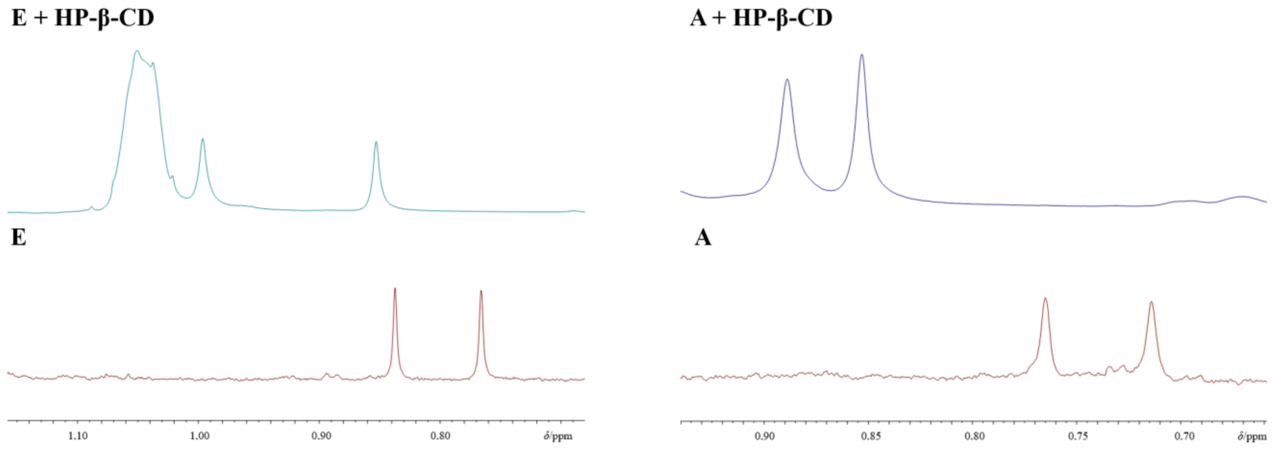

Figure S4. Selected regions of the ^{1}H NMR spectra of solutions of **E** and **A** and of their mixtures with **HP-β-CD** in D_2O at 298 K. The broad signal at $\delta \approx 1.05$ ppm corresponds to the receptor.

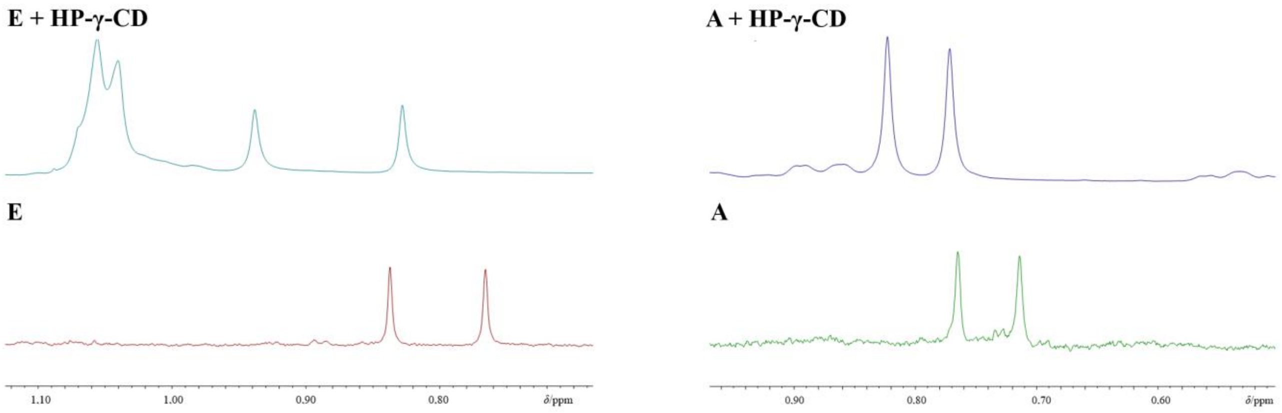

Figure S5. Selected regions of the ^{1}H NMR spectra of solutions of **E** and **A** and of their mixtures with **HP-γ-CD** in D_2O at 298 K. The broad signal at $\delta \approx 1.05$ ppm corresponds to the receptor.

S1.2. Randomly methylated β-cyclodextrin

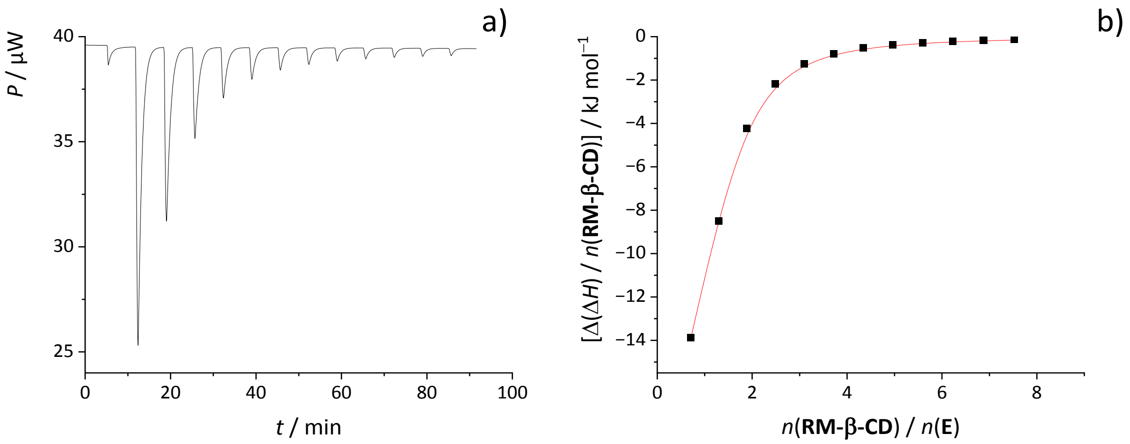

Figure S6. Microcalorimetric titration of **E** (c_0 = 7.40 × 10^{-5} mol dm^{-3}, V = 1.45 cm^3) with **RM-β-CD** (c = 5.02 × 10^{-3} mol dm^{-3}) in H_2O at 298 K. (a) Thermogram. (b) Dependence of the normalized successive enthalpy change on the host-to-guest ratio. ■ experimental; – calculated.

S1.3. Sulfobutylether-β-cyclodextrin

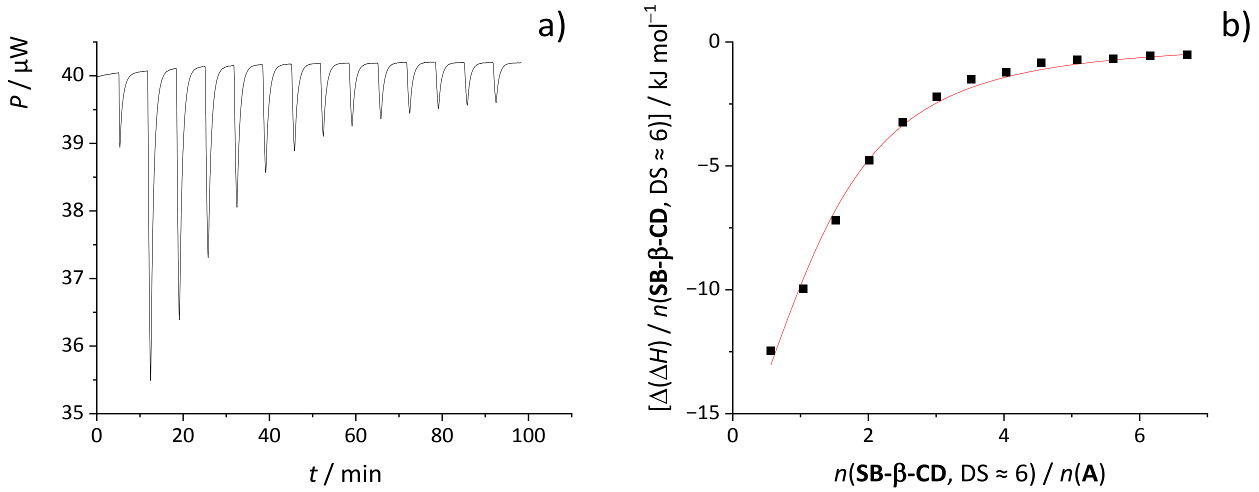

Figure S7. Microcalorimetric titration of **A** (c_0 = 3.06 × 10^{-5} mol dm^{-3}, V = 1.45 cm^3) with **SB-β-CD** (DS ≈ 6, c = 9.98 × 10^{-4} mol dm^{-3}) in H_2O at 298 K. (a) Thermogram. (b) Dependence of the normalized successive enthalpy change on the host-to-guest ratio. ■ experimental; – calculated.

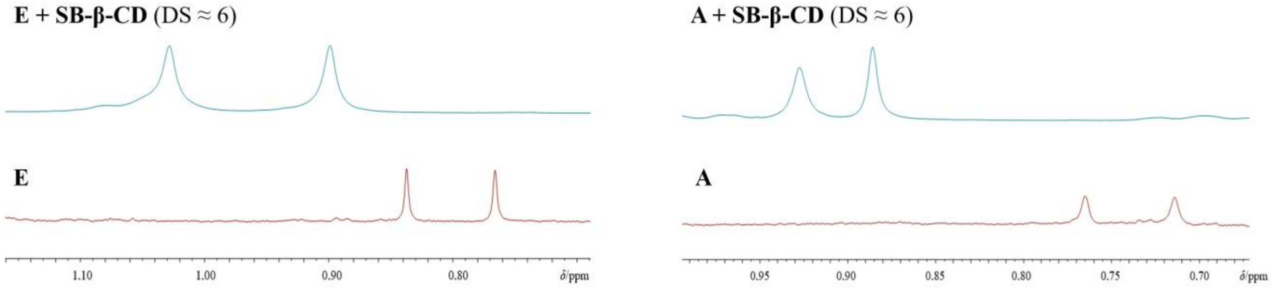

Figure S8. Selected regions of the ^{1}H NMR spectra of solution of **E** and **A** and of their mixtures with **SB-β-CD** (DS ≈ 6) in D_2O at 298 K.

S1.4. Influence of receptor substitution on *trans*-steroid solubility

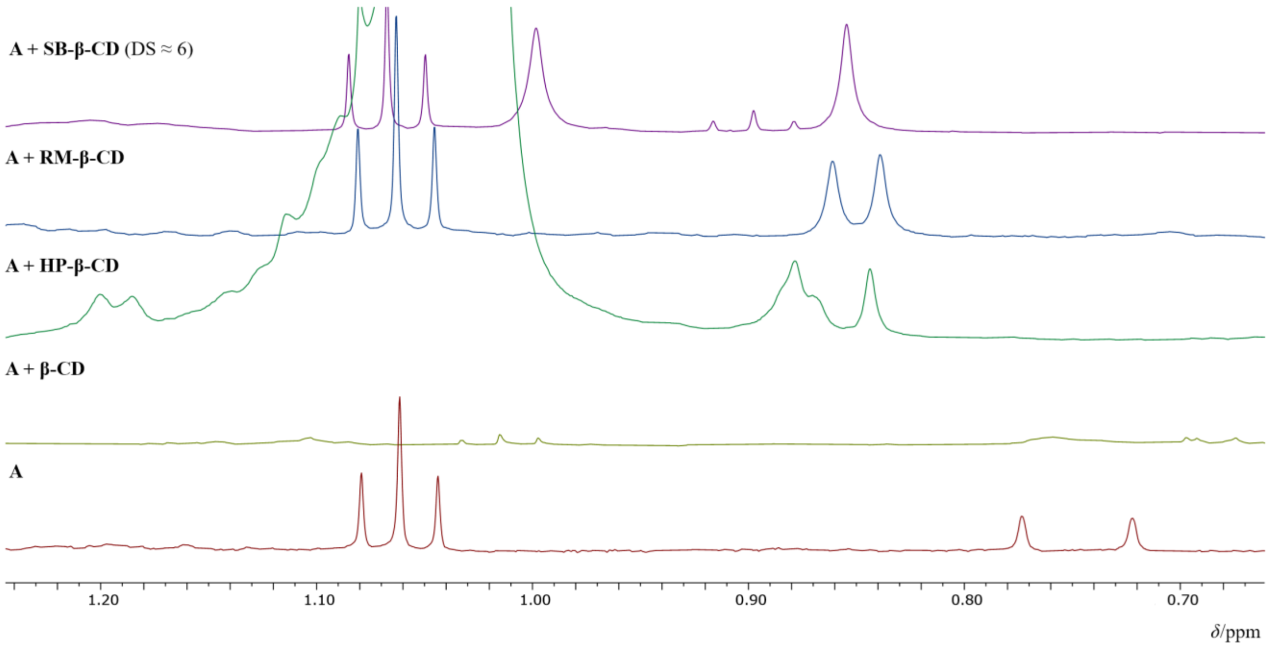

Figure S9. Selected regions of the ^{1}H NMR spectra of **A** (suspension prepared by dissolving 0.35 mg in 10 cm^3 of D_2O to obtain c(**A**) = 1.2×10^{-4} mol dm^{-3}) and its mixtures containing 10 equivalents of **β-CD**, **HP-β-CD**, **RM-β-CD**, and **SB-β-CD** (DS ≈ 6). Samples were prepared by adding 0.10 cm^3 stock receptor solution (c = 6.0×10^{-3} mol dm^{-3}) to 0.50 cm^3 suspension of **A** and 0.10 cm^3 D_2O was added to the suspension of **A** to avoid the effect of dilution by receptor solution. All spectra were recorded at 298 K. All samples with the exception of **A** + **β-CD** became clear upon addition of receptor solution due to formation of highly soluble **A**:receptor complexes with substituted cyclodextrins. Visibly smaller and broader signals corresponding to **A** and its complexes with **β-CD** are due to previously observed precipitation of **A**:(**β-CD**)$_2$ complex (see Požar, J. *et al.* Molecular Recognition of Steroid Isomers by Cyclodextrins and Cucurbiturils: Thermodynamic Contributions and Structural Determinants. *New J. Chem*. **2026**, doi:10.1039/D6NJ02208A)

S2. Complexation of bile salts with substituted cyclodextrins

S2.1. 2-Hydroxypropylated cyclodextrins

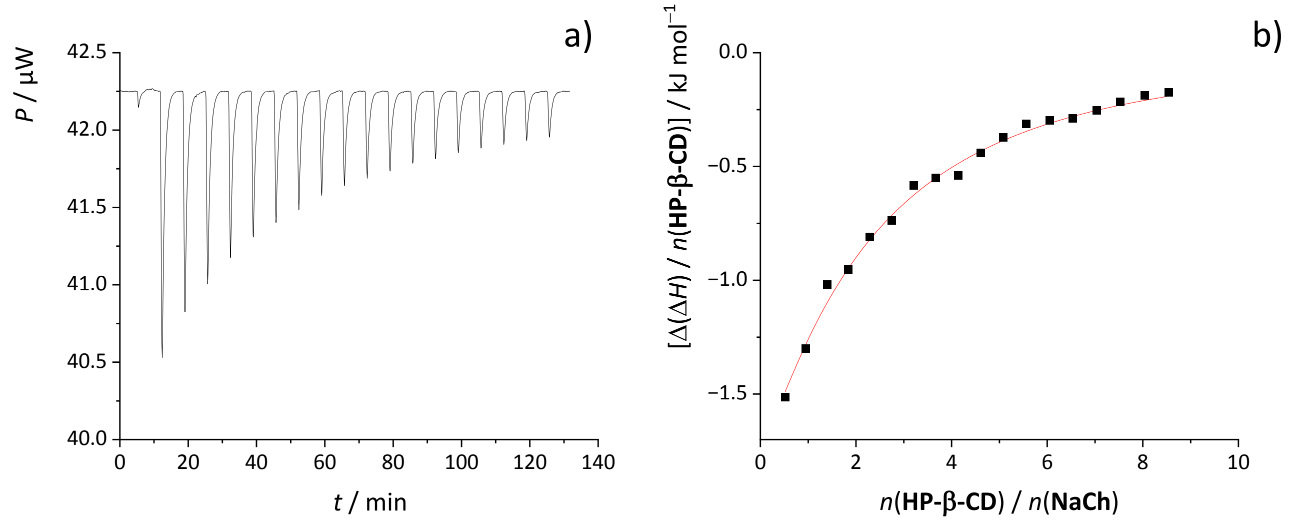

Figure S10. Microcalorimetric titration of **NaCh** ($c_0 = 1.09 \times 10^{-4}$ mol dm^{-3}, $V = 1.45$ cm^3) with **HP-β-CD** ($c = 4.51 \times 10^{-3}$ mol dm^{-3}) in H_2O at 298 K. (a) Thermogram. (b) Dependence of the normalized successive enthalpy change on the host-to-guest ratio. ■ experimental; − calculated.

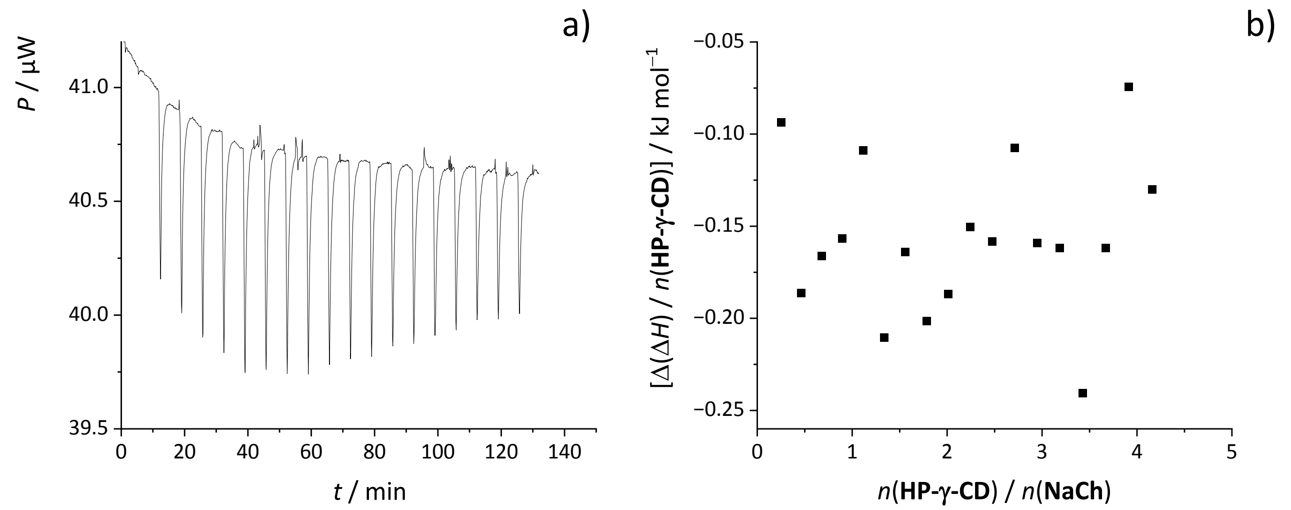

Figure S11. Microcalorimetric titration of **NaCh** ($c_0 = 5.48 \times 10^{-4}$ mol dm^{-3}, $V = 1.45$ cm^3) with **HP-γ-CD** ($c = 1.11 \times 10^{-2}$ mol dm^{-3}) in H_2O at 298 K. (a) Thermogram. (b) Dependence of the normalized successive enthalpy change on the host-to-guest ratio.

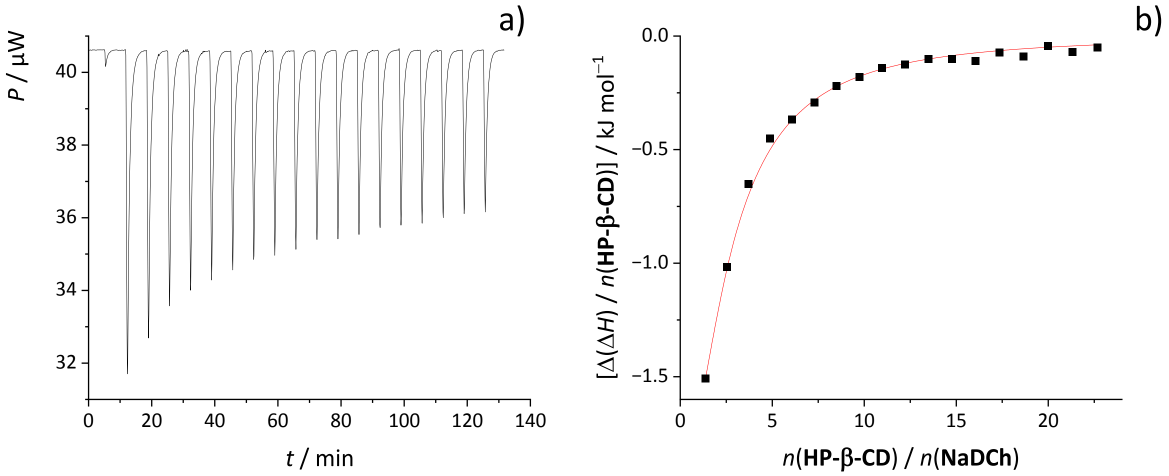

Figure S12. Microcalorimetric titration of **NaDCh** (c_0 = 9.10 × 10^{-5} mol dm^{-3}, V = 1.45 cm^3) with **HP-β-CD** (c = 1.00 × 10^{-2} mol dm^{-3}) in NaOH (c = 5.11 × 10^{-3} mol dm^{-3}) at 298 K. (a) Thermogram. (b) Dependence of the normalized successive enthalpy change on the host-to-guest ratio. ■ experimental; – calculated.

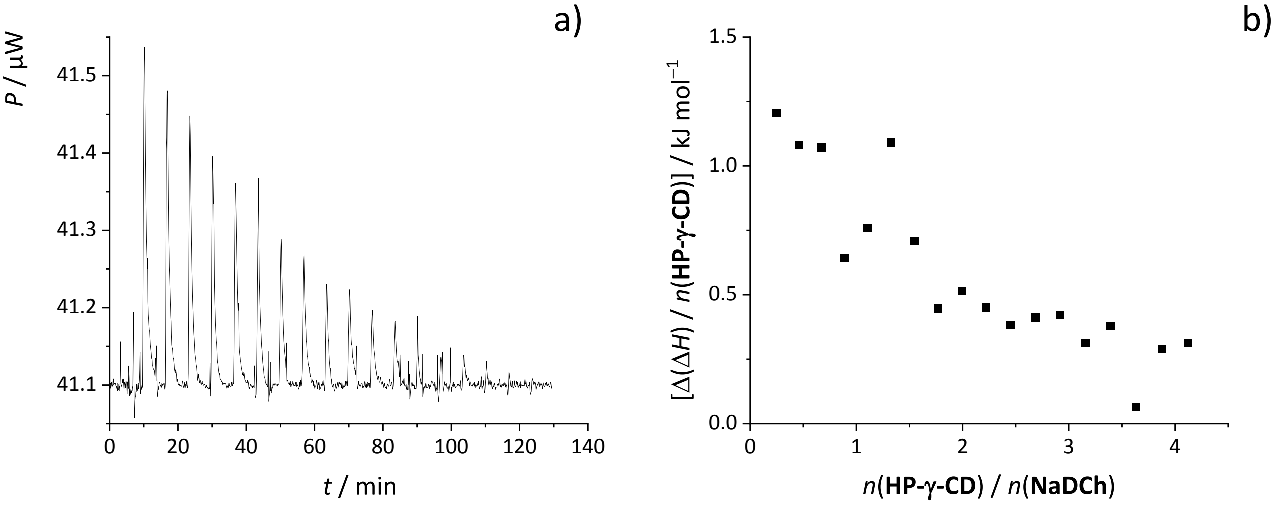

Figure S13. Microcalorimetric titration of **NaDCh** (c_0 = 9.10 × 10^{-5} mol dm^{-3}, V = 1.45 cm^3) with **HP-γ-CD** (c = 1.82 × 10^{-3} mol dm^{-3}) in NaOH (c = 5.11 × 10^{-3} mol dm^{-3}) at 298 K. (a) Thermogram. (b) Dependence of the normalized successive enthalpy change on the host-to-guest ratio.

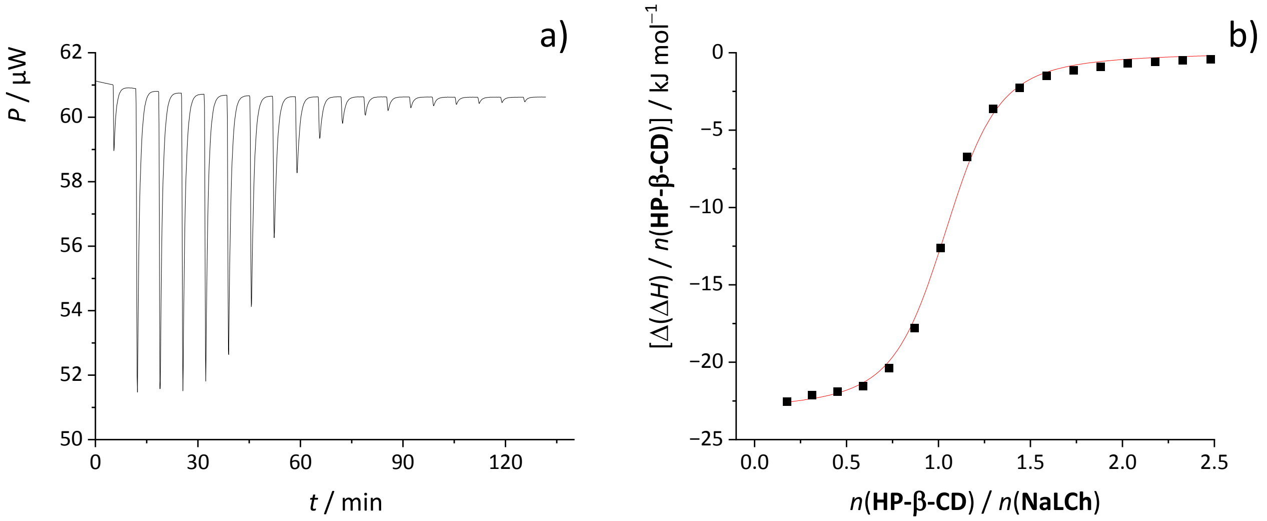

Figure S14. Microcalorimetric titration of **NaLCh** (c_0 = 1.22 × 10^{-4} mol dm^{-3}, V = 1.45 cm^3) with **HP-β-CD** (c = 1.11 × 10^{-3} mol dm^{-3}) in NaOH (c = 5.01 × 10^{-3} mol dm^{-3}) at 298 K. (a) Thermogram. (b) Dependence of the normalized successive enthalpy change on the host-to-guest ratio. ■ experimental; – calculated.

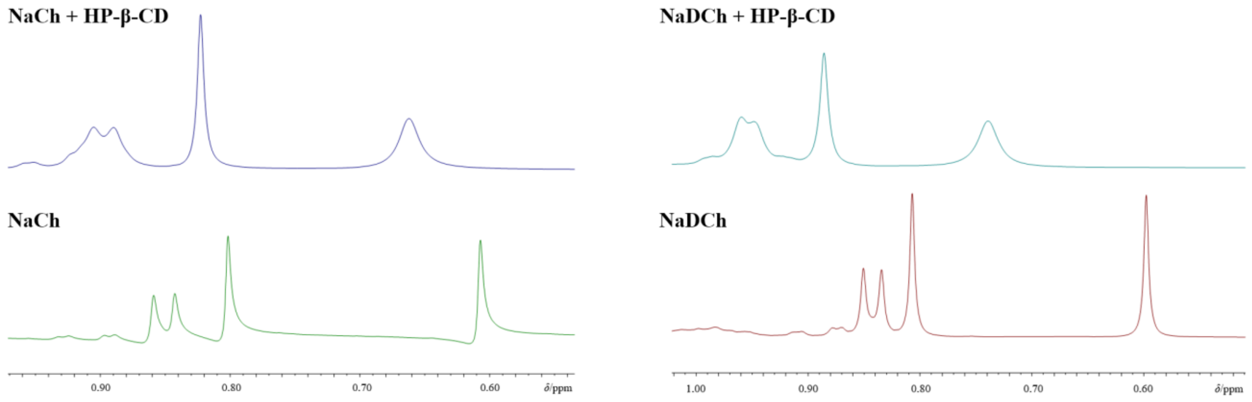

Figure S15. Selected regions of the ^{1}H NMR spectra of solutions of **NaCh** and **NaDCh** and of their mixtures with **HP-β-CD** in D_2O at 298 K.

S2.2. Sulfobutylether β-cyclodextrin

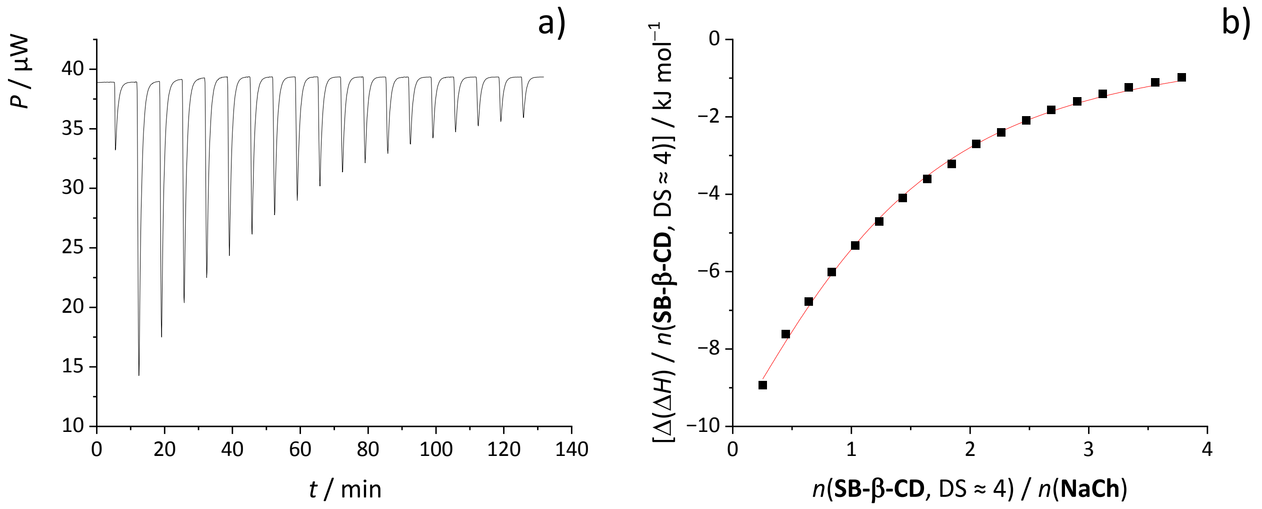

Figure S16. Microcalorimetric titration of **NaCh** ($c_0 = 5.48 \times 10^{-4}$ mol dm^{-3}, $V = 1.45$ cm^3) with **SB-β-CD** (DS ≈ 4, $c = 9.98 \times 10^{-3}$ mol dm^{-3}) in H_2O at 298 K. (a) Thermogram. (b) Dependence of the normalized successive enthalpy change on the host-to-guest ratio. ■ experimental; – calculated.

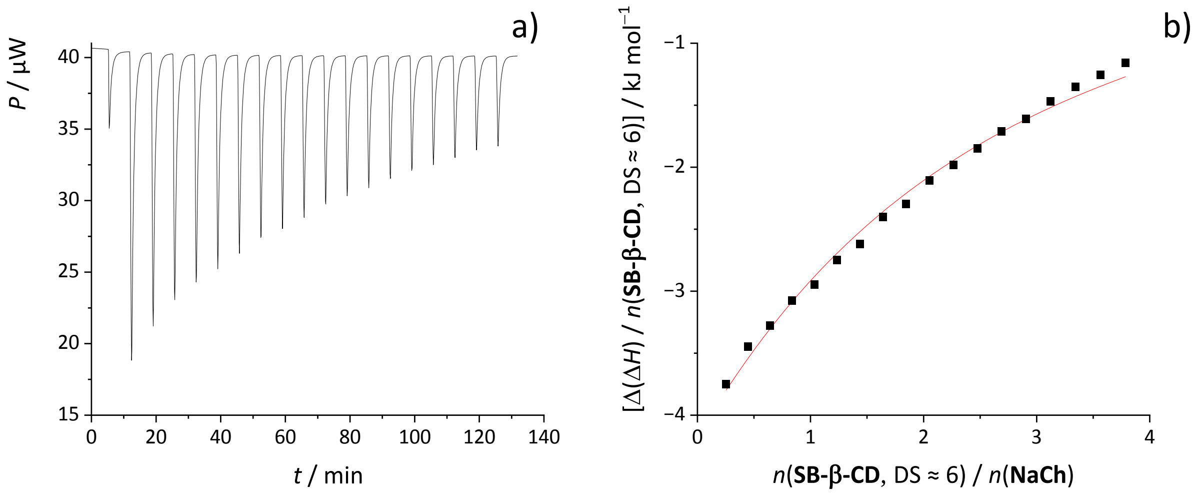

Figure S17. Microcalorimetric titration of **NaCh** ($c_0 = 5.48 \times 10^{-4}$ mol dm^{-3}, $V = 1.45$ cm^3) with **SB-β-CD** (DS ≈ 6, $c = 9.99 \times 10^{-3}$ mol dm^{-3}) in H_2O at 298 K. (a) Thermogram. (b) Dependence of the normalized successive enthalpy change on the host-to-guest ratio. ■ experimental; – calculated.

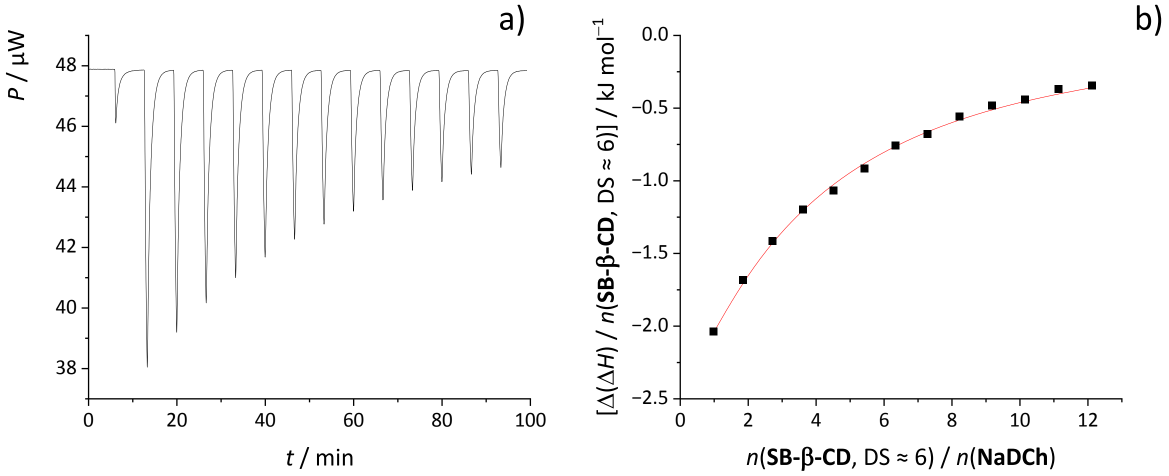

Figure S18. Microcalorimetric titration of **NaDCh** (c_0 = 1.15 × 10^{-4} mol dm^{-3}, V = 1.45 cm^3) with **SB-β-CD** (DS ≈ 6, c = 6.96 × 10^{-3} mol dm^{-3}) in NaOH (c = 5.11 × 10^{-3} mol dm^{-3}) at 298 K. (a) Thermogram. (b) Dependence of the normalized successive enthalpy change on the host-to-guest ratio. ■ experimental; − calculated.

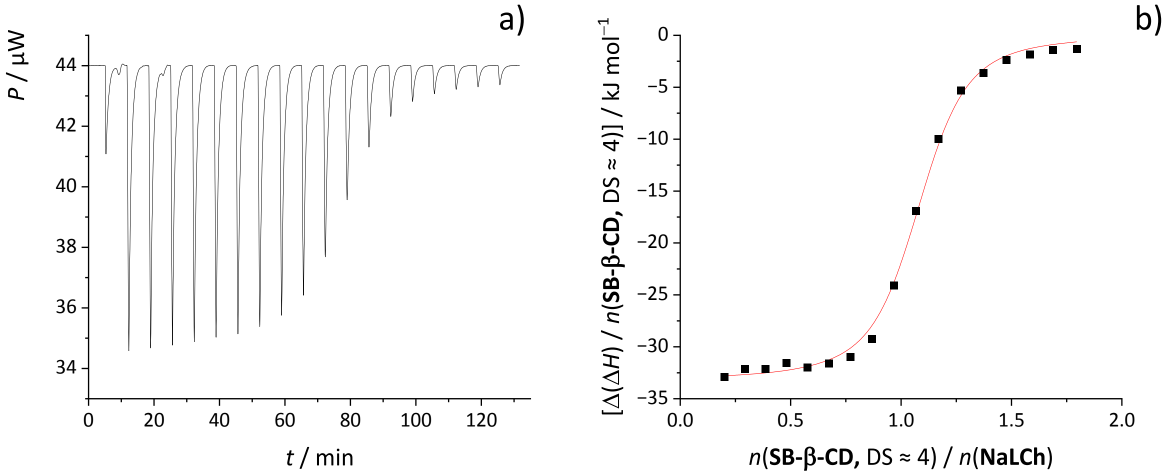

Figure S19. Microcalorimetric titration of **NaLCh** (c_0 = 1.22 × 10^{-4} mol dm^{-3}, V = 1.45 cm^3) with **SB-β-CD** (DS ≈ 4, c = 1.11 × 10^{-3} mol dm^{-3}) in NaOH (c = 5.01 × 10^{-3} mol dm^{-3}) at 298 K. (a) Thermogram. (b) Dependence of the normalized successive enthalpy change on the host-to-guest ratio. ■ experimental; − calculated.

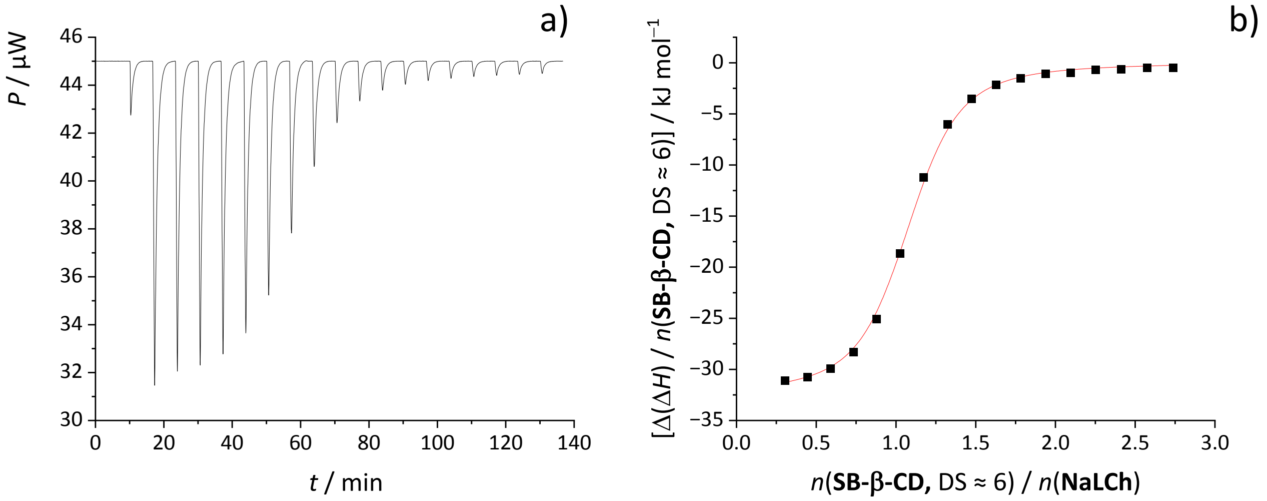

Figure S20. Microcalorimetric titration of **NaLCh** ($c_0 = 1.22 \times 10^{-4}$ mol dm^{-3}, $V = 1.45$ cm^3) with **SB-β-CD** (DS ≈ 6, $c = 1.60 \times 10^{-3}$ mol dm^{-3}) in NaOH ($c = 5.01 \times 10^{-3}$ mol dm^{-3}) at 298 K. (a) Thermogram. (b) Dependence of the normalized successive enthalpy change on the host-to-guest ratio. ■ experimental; − calculated.

Table S1. Thermodynamic complexation parameters of **NaDCh** with **β-CD** in H_2O and in NaOH(aq) ($c_{NaOH} = 5 \times 10^{-3}$ mol dm^{-3}) at 298 K.[a]

Solvent	log $K°$	$\Delta_r G°$ / kJ mol^{-1}	$\Delta_r H°$ / kJ mol^{-1}	$-T\Delta_r S°$ / kJ mol^{-1}
H_2O	3.60(1)	−20.54(5)	−33.8(6)	13.2(6)
NaOH(aq)	3.85(1)	−21.99(3)	−34.6(1)	12.6(1)

[a] Standard error of the mean (N = 3–5) is given in parentheses as the uncertainty of the last significant digit.
